# 1,3-Alternate Calix[4]arene Functionalized With Pyrazole and Triazole Ligands as a Highly Selective Fluorescent Sensor for Hg^2+^ and Ag^+^ Ions

**DOI:** 10.3389/fchem.2020.593261

**Published:** 2020-11-12

**Authors:** Yin-Ju Chen, Meng-Yu Chen, Kun-Ti Lee, Li-Ching Shen, Hao-Chih Hung, Hao-Che Niu, Wen-Sheng Chung

**Affiliations:** Department of Applied Chemistry, National Chiao Tung University, Hsinchu, Taiwan

**Keywords:** fluorescent sensor, mercury (II) sensor, silver (I) sensor, homoditropic, 1, 3-alternate calix[4]arene, pyrazole, triazole

## Abstract

We report here the synthesis of a 1,3-alternate calix[4]arene **8**, with bis-pyrazolylmethylpyrenes on the one end and bis-triazolylmethylphenyls on the other end, as a homoditropic fluorescent sensor for both Hg^2+^ and Ag^+^ ions. Calix[4]arene **3**, with lower-rim bis-pyrazolylmethylpyrenes in cone conformation, was also synthesized as a control compound. UV-Vis and fluorescence spectra were used for metal ions screening, and we found that both ligands **8** and **3** showed strong excimer emission of pyrenes when they are as a free ligand in CHCl_3_/MeOH (v/v, 3:1) solution; however, they both showed a high selectivity toward Hg^2+^ and Ag^+^ ions with strong fluorescence quenching and yet with different binding ratios. The fluorescence of ligand **8** was strongly quenched by Hg^2+^ but was only partially quenched by Ag^+^ ions; however, the fluorescence of ligand **3** was strongly quenched by Hg^2+^, Ag^+^, and Cu^2+^ ions. Job plot experiments showed that ligand **8** formed a 1:2 complex with both Hg^2+^ and Ag^+^ ions; ligand **3** formed a 1:1 complex with Hg^2+^, but it formed a 2:3 complex with Ag^+^. The binding constant of ligand **3** with Hg^2+^ and Ag^+^ ions was determined by the Benesi-Hildebrand plot of UV-vis titration experiments to be 2.99 × 10^3^ and 3.83 × 10^3^ M^−1^, respectively, while the association constant of ligand **8** with Hg^2+^ and Ag^+^ was determined by Hill plot to be 1.46 × 10^12^ and 9.24 × 10^11^ M^−2^, respectively. Ligand **8** forms a strong complex with either two Hg^2+^ or two Ag^+^ ions using both the upper and lower rims of the 1,3-alternate calix[4]arene as the binding pockets; hence, it represents one of the highly selective fluorescent sensors for the homoditropic sensing of Hg^2+^ and Ag^+^ ions.

## Introduction

Over the past two decades, the design and synthesis of fluorescent chemosensors for the selective sensing of heavy and transition-metal (HTM) ions have attracted much attention in the fields of supramolecular chemistry, biology, and medicinal chemistry (Czarnik, 1997; de Silva et al., 1997; Prodi et al., 2000; Valeur and Leray, 2000; Li et al., 2014). Accumulation of HTM ions in the bodies of humans and animals can lead to serious illnesses even in low concentration. Mercury has been regarded as one of the most toxic HTM ions, and it exists in a variety of different forms. Mercury rapidly bioaccumulates in the physiological functionalities due to microbial conversion of its elemental and ionic forms into methyl mercury (Poulain and Barkay, 2013), which subsequently passes through skin, respiratory, and gastrointestinal tissues into the human body. Exposure to mercury leads to dysfunction of cells and consequently causes many health problems in the brain, kidney, and central nervous, mitosis, and endocrine systems because of its high affinity for thiol groups in proteins and enzymes (Wang et al., 2011, Bai et al., 2020). Meanwhile, silver ion is also known to have negative impacts on the environment and human beings; for example, it inactivates sulfhydryl enzymes and binds with various metabolites. However, one should know that Ag^+^ has moderate coordination ability, making it quite difficult to be discriminated from other chemically similar heavy transition metal ions (Wang et al., 2012). Therefore, considerable effort has been made to the design and synthesis of fluorescent probes that can selectively and sensitively detect Hg^2+^ and Ag^+^ ions (Ho et al., 2011; Wang et al., 2011, 2012; Kim et al., 2012).

As part of our continuous interest in the design and synthesis of novel fluorescent chemosensors (Chang et al., 2007, 2008; Hung et al., 2009a,b; Senthilvelan et al., 2009; Ho et al., 2011; Wang et al., 2011, 2012), we utilize here the 1,3-alternate calix[4]arene platform to construct a ditopic fluorescent sensor **8**, which contains bis-pyrazoles on the one rim and bis-triazoles on the other rim as metal ion binding sites and pyrene pendants as fluorophores (reporters). Pyrene is one of the most useful fluorogenic units because it exhibits high fluorescence quantum yield, and it emits not only from the monomer but also from the excimer (Winnik, 1993; Yang et al., 2001; Hung et al., 2014). Many fluorescent chemosensors using pyrene as a fluorophore have been reported; for example, Kim et al. (2005) reported a calix[4]arene-based fluorescent sensor with two fluorogenic pyrene units conjugated to amide groups as guest recognition sites. When complexed with fluoride anion, their compound showed a big bathochromic shift on its absorption band, but the excimer emission was blue shifted to 470 nm (Δλ = 12 nm) together with an enhanced fluorescence intensity. In 2008, Kim and Yang independently reported (Park et al., 2008; Zhu et al., 2008) the synthesis of a calix[4]arene with two lower-rim pyrene units linked with triazole groups, which showed ratiometric fluorescence response in acetonitrile solution toward Zn^2+^ and Cd^2+^ and selective fluorescence quenching toward HTM ions such as Cu^2+^, Hg^2+^, and Pb^2+^.

Pyrazoles have been frequently utilized as a ligand in coordination chemistry (Mukherjee, 2000; Viciano-Chumillas et al., 2010), for example, Kumar et al. reported that biscalix[4]arenes having pyrazoles as bridges behaved as a highly selective ionophore toward silver ions (Kumar et al., 2003). Elanchezhian and Kandaswamy also reported a ferrocene-pyrazole as an on–off type fluorescence chemosensor for Cu^2+^, while it also behaved as a Hg^2+^ selective fluorescent sensor that showed bathochromic shift after complexation (Elanchezhian and Kandaswamy, 2010). Triazoles, which can be readily synthesized by click chemistry, have been popularly used as ligands in coordination chemistry (Rostovtsev et al., 2002; Chang et al., 2007; Colasson et al., 2007; Park et al., 2008; Zhu et al., 2008; Hung et al., 2009b; Zhan et al., 2009; Gower and Crowley, 2010; Hua and Flood, 2010; Kim et al., 2010; Le Droumaguet et al., 2010; Ho et al., 2011; Lau et al., 2011). For example, we have demonstrated that a triazole-modified calix[4]crown in 1,3-alternate conformation can behave as a Pb^2+^/K^+^ off–on switchable fluorescent chemosensor (Chang et al., 2007). In another study, we also showed that a 1,3-alternate calix[4]arene, containing bis-triazoles on the one rim and bis-enaminone groups on the other rim, functioned as a homoditropic fluorescent sensor for two Ag^+^ ions (Ho et al., 2011). Despite the fact that pyrazoles and triazoles are both popular ligands in coordination chemistry, their relative metal ion sensing abilities have not been assessed. As such, we report here the synthesis of 1,3-alternate calix[4]arene **8**, which contains bis-pyrazolylmethyphenyls on the one rim and bis-triazolylmethylpyrenes on the other rim, as a highly selective fluorescent sensor for Hg^2+^ and Ag^+^ ions. Calix[4]arene **3**, which contains bis-triazolyl-methylpyrenes in cone conformation, was also synthesized to compare with compound **8** for their metal ion selectivity.

## Materials and Physical Methods

### Synthesis of 25,27-Bisoxy-(3-Methyl-1-Pyren-1-Ylmethyl-1H-Pyrazole)-26,28-Dihydroxy-Calix[4]arene 3

A mixture of calix[4]arene **1** (1.56 g, 3.7 mmol), 3-(bromomethyl)-1-(pyren-1-ylmethyl)-1*H*-pyrozole **2** (2.75 g, 7.3 mmol), and K_2_CO_3_ (1.00 g, 7.3 mmol) in MeCN was stirred at reflux for 20 h. The solvent was removed by reduced pressure. The residue obtained after evaporation of the solvent was subjected to a silica gel flash column chromatography using a gradient polarity (EtOAc/*n*-hexane = 1:5 to 1:2) to afford **3** (1.88 g, 51%). mp 178–180°C. ^1^H NMR (CDCl_3_, 400 MHz): δ 8.12–8.05 (m, 6H), 7.98–7.88 (m, 10H), 7.81 (s, 2H), 7.54 (d, *J* = 7.8 Hz, 2H), 7.01 (d, *J* = 7.5 Hz, 4H), 6.85 (d, *J* = 7.5 Hz, 4H), 6.74–6.61 (m, 4H), 6.63 (t, *J* = 7.5 Hz, 2H), 6.42 (d, *J* = 2.3 Hz, 2H), 5.72 (s, 4H), 5.07 (s, 4H), 4.31 (d, *J* = 13.1 Hz, 4H), 3.28 (d, *J* = 13.2 Hz, 4H) (Supplementary Figure 1, ESI). ^13^C NMR (CDCl_3_, 100 MHz): δ 153.3 (Cq), 151,7 (Cq), 148.3 (Cq), 133.4 (Cq), 131.5 (Cq), 131.1 (Cq), 130.6 (Cq), 130.1 (CH), 128.9 (Cq), 128.9 (CH), 128.7 (Cq),128.5 (CH), 128.4 (CH), 128.1 (Cq), 127.7 (CH), 127.2 (CH), 126.9 (CH), 126.1 (CH), 125.5 (CH), 125.5 (CH), 125.3 (CH), 124.8 (CH), 124.7 (CH), 124.4 (Cq), 122.3 (CH), 118.8 (CH), 106.3 (CH), 72.2 (CH_2_), 54.0 (CH_2_), 31.4 (CH_2_) (Supplementary Figure 2, ESI). HRMS (FAB): calcd for C_70_H_53_N_4_O_4_ [M + H^+^] 1,013.4067; found 1,013.4043. Calcd for C_70_H_52_AgN_4_O_4_ [M + Ag^+^] 1,119.3039; found 1,119.3054 (Supplementary Figure 9e, ESI).

### Synthesis of 1,3-Alternate Calix[4]arene 5

A mixture of 25,27-dipropargyloxy-26,28-dihydroxycalix[4]arene **6** (Chang et al., 2007) (0.27 g, 0.55 mmol), 3-(bromomethyl)-1-(pyren-1-ylmethyl)-1*H*-pyrazole **2** (0.43 g, 1.15 mmol), and Cs_2_CO_3_ (1.79 g, 5.49 mmol) was in tetrahydrofuran (THF) stirred at reflux for 22 h. The solvent was removed by reduced pressure. The residue obtained after evaporation of the solvent was subjected to a silica gel flash column chromatography using a gradient polarity (EtOAc/*n*-hexane = 1:3 to 2:1) to afford **5** (0.36 g, 60%). White yellow solid; *R*_f_ = 0.25 (EtOAc/*n*-hexane = 1:2); mp 185–187°C. ^1^H NMR (CDCl_3_, 400 MHz): δ 8.18–7.93 (m, 17H), 7.74 (d, *J* = 7.8 Hz, 2H), 7.14 (d, *J* = 7.5 Hz, 4H), 6.98 (d, *J* = 1.7 Hz, 2H), 6.86–6.80 (m, 6H), 6.37 (t, *J* = 7.5 Hz, 2H), 5.91 (s, 4H), 5.85 (d, *J* = 2.0 Hz, 2H), 4.83 (s, 4H), 3.98 (d, *J* = 2.2 Hz, 4H), 3.73 (d, *J* = 14.5 Hz, 4H), 3.63 (d, *J* = 14.5 Hz, 4H), 2.42 (s, 2H) (Supplementary Figure 3, ESI). ^13^C NMR (CDCl_3_, 100 MHz): δ 155.9 (Cq), 155.1 (Cq), 149.6 (Cq), 134.1 (Cq), 133.9 (Cq), 131.5 (Cq), 131.1 (Cq), 130.6 (Cq), 130.5 (CH), 130.2 (CH), 129.5 (CH), 129.0 (Cq), 128.9 (Cq), 128.5 (CH), 127.7 (CH), 127.2 (CH), 127.1 (CH), 126.1 (CH), 125.5 (CH), 125.4 (CH), 124.9 (Cq), 124.8 (CH), 124.5 (Cq), 122.7 (CH), 122.6 (CH), 122.3 (CH), 106.6 (CH), 80.6 (Cq), 74.4 (Cq), 67.1 (CH_2_), 58.9 (CH_2_), 54.1 (CH_2_), 36.9 (CH_2_) (Supplementary Figure 4, ESI). HRMS (FAB) *m*/*z*: calcd for C_76_H_58_N_4_O_4_ [M]^+^ 1,090.4458; found 1,090.4468.

### Synthesis of 1,3-Alternate Calix[4]arene 8

A mixture of calix[4]arene **5** (0.25 g, 0.229 mmol), azidomethylbenzene **7** (0.085 ml, 0.688 mmol), Cu_2_SO_4_.H_2_O (0.03 g, 0.115 mmol) and sodium ascorbate (0.03 g, 0.115 mmol) was in THF/H_2_O (6:1, v/v) stirred at reflux for 24 h. The solvent was removed by reduced pressure. The residue obtained after evaporation of the solvent was subjected to a silica gel flash column chromatography using a gradient polarity (EtOAc/*n*-hexane = 1:3 to 2:1) to afford **8** (192 mg, 62%). White yellow solid; *R*_f_ = 0.25 (EtOAc/*n*-hexane = 1:1); mp 210–212°C. ^1^H NMR (CDCl_3_, 600 MHz): δ 8.16–7.86 (m, 16H), 7.62 (d, *J* = 7.7 Hz, 2H), 7.44–7.31 (m, 6H), 7.30–7.26 (m, 4H), 6.77 (d, *J* = 2.0 Hz, 2H), 6.71 (d, *J* = 7.5 Hz, 4H), 6.63 (d, *J* = 7.5 Hz, 4H), 6.60 (s, 2H), 6.28 (t, *J* = 7.5 Hz, 2H), 6.09 (t, *J* = 7.5 Hz, 2H), 5.75 (s, 4H), 5.56 (s, 4H), 5.50 (d, *J* = 2.0 Hz, 2H), 4.70 (s, 4H), 4.68 (s, 4H), 3.58 (d, *J* = 15.4 Hz, 4H), 3.39 (d, *J* = 15.4 Hz, 4H) (Supplementary Figure 5, ESI). ^13^C NMR (CDCl_3_, 150 MHz): δ 156.0 (Cq), 154.9 (Cq), 149.6 (Cq), 145.1 (Cq), 133.9 (Cq), 133.8 (Cq), 131.5 (Cq), 131.1 (Cq), 130.6 (Cq), 129.8 (CH), 129.25 (CH), 129.3 (CH), 129.0 (Cq), 128.9 (Cq), 128.5 (CH), 128.4 (CH), 127.8 (CH), 127.7 (CH), 127.2 (CH), 127.0 (CH), 126.1 (CH), 125.6 (CH), 125.5 (CH), 124.9 (CH), 124.7 (Cq), 124.5 (Cq), 123.4 (Cq), 122.9 (CH), 122.3 (CH), 122.1 (CH), 106.6 (CH), 66.2 (CH_2_), 63.9 (CH_2_), 53.8 (CH_2_), 53.7 (CH_2_), 37.5 (CH_2_) (Supplementary Figure 6, ESI). ESI: *m/z* 1,357 (M)^+^, 1,463 (M + Ag^+^), 1,557 (M + Hg^+^). HRMS: *m/z* calcd for C_90_H_70_AgN_10_O_4_ (M + Ag^+^) 1,461.4632, found 1,461.4713. Calcd for [C_90_H_70_Ag_2_N_10_O_4_]^2+^•[${\text{ClO}}_{4}^{-}$] (M + 2Ag^+^ + ${\text{ClO}}_{4}^{-}$) 1,667.3168, found 1,667.3253 (Supplementary Figure 18, ESI).

### General Procedures for the UV/Vis and Fluorescence Experiments

UV-vis spectra were recorded on a spectrophotometer with a diode array detector, and the resolution was set at 1 nm. Fluorescence spectra were recorded on a luminescence spectrophotometer. For all measurements of fluorescence spectra, excitation was set at 345 nm for compound **3** and at 347 nm for 1,3-alternate **8**; the excitation and emission slit width was set to be 4.0 nm. UV-vis and fluorescence titration experiments were performed with 10 μM solutions of compounds **3** and **8** and varying concentrations of metal perchlorate in CHCl_3_/MeOH (v/v, 3:1) co-solvent. During all measurements, the temperature of the quartz sample cell and chamber was kept at 25°C.

### General Procedures for the ^1^H-NMR Titration Experiments

^1^H-NMR titration spectra were recorded at 300 or 600 MHz (variable temperature experiments) with tetramethylsilane (TMS) in CDCl_3_ in a coaxial capillary tube as an external standard. Experiments were performed with 1.33 mM solutions of compound **3** or **8** in CDCl_3_/CD_3_OH (v/v, 3:1) co-solvent by adding various concentrations of AgClO_4_ or Hg(ClO_4_)_2_ at 25°C.

## Results and Discussion

**Scheme 1:**
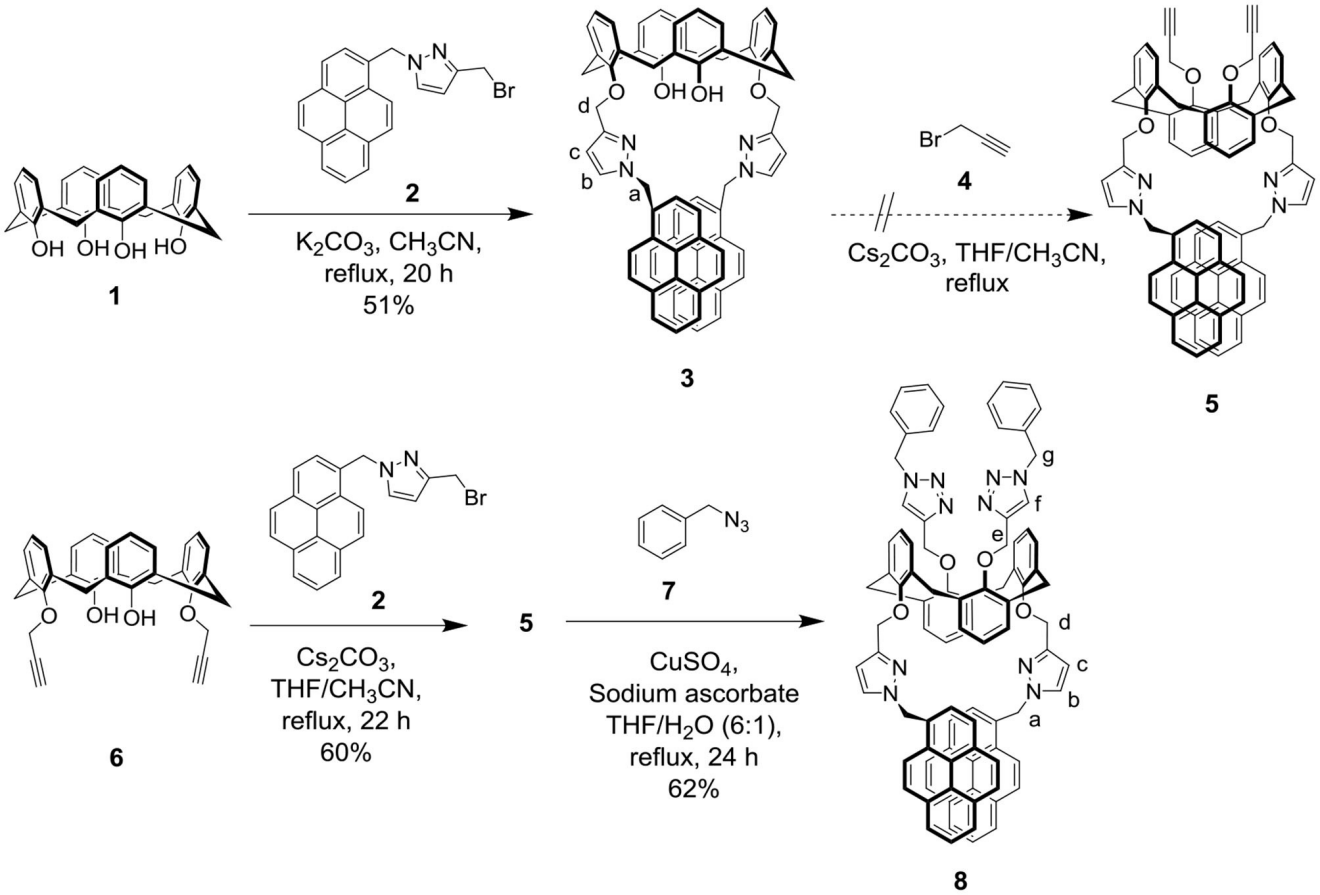
Syntheses of target molecule **8** and control compound **3**.

The syntheses of target molecule **8** and control compound **3** are depicted in Scheme 1. Calix[4]arene **1** was reacted with 3-(bromomethyl)-1-(pyren-1-ylmethyl)-1*H*-pyrazole **2** (which requires a four-step synthesis from a commercial compound, **Scheme S1**, ESI) under basic conditions to generate 25,27-bisoxy-(3-methyl-1-pyren-1-ylmethyl-1*H*-pyrazole)-26,28-dihydroxycalix[4]arene **3** in 51% yield. The methylene bridge carbon atoms (Ar*C*H_2_Ar) of calix[4]arene **3** that appeared at 31.4 ppm in the ^13^C NMR is characteristic of a cone conformation of calix[4]arene (Supplementary Figure 2, ESI) (Shiao et al., 2006). Further etherification of compound **3** with propargyl bromide **4** in the presence of Cs_2_CO_3_ failed to afford the 1,3-alternate calix[4]arene **5**; however, under similar reaction conditions, bispropargylether substituted calix[4]arene **6 (**Chang et al., 2007) successfully reacted with pyrazole bromide **2** to furnish the 1,3-alternate calix[4]arene **5** in 60% yield. The 1,3-alternate conformation of **5** was supported by the following observations: (1) its methylene bridge protons (ArC*H*_2_Ar) appeared as an AB quartet at 3.72 and 3.62 ppm in ^1^H NMR and (2) the four methylene bridge carbon atoms (Ar*C*H_2_Ar) appeared as a single peak at 36.9 ppm in the DEPT ^13^C NMR spectra (Supplementary Figures 3, 4, ESI) (Jaime et al., 1991; Mandolini and Ungaro, 2000). Under click chemistry conditions, the azidomethylbenzene **7** reacted with the two propargyl groups of **5** to form bisphenylmethyltriazole substituted 1,3-alternate calix[4]arene **8** in 60%. The structure of compound **8** was confirmed by the appearance of its triazole protons (H-f) at 6.60 ppm and its triazole carbons (C-f) at 122.9 ppm, respectively, in ^1^H and DEPT ^13^C NMR spectra (Supplementary Figures 5, 6, ESI). All calix[4]arene compounds **3**, **5**, and **8** were fully characterized by ^1^H and DEPT ^13^C NMR spectroscopy, mass spectrometry, and high-resolution mass spectrometry (see section Materials and Physical Methods).

**Figure 1:**
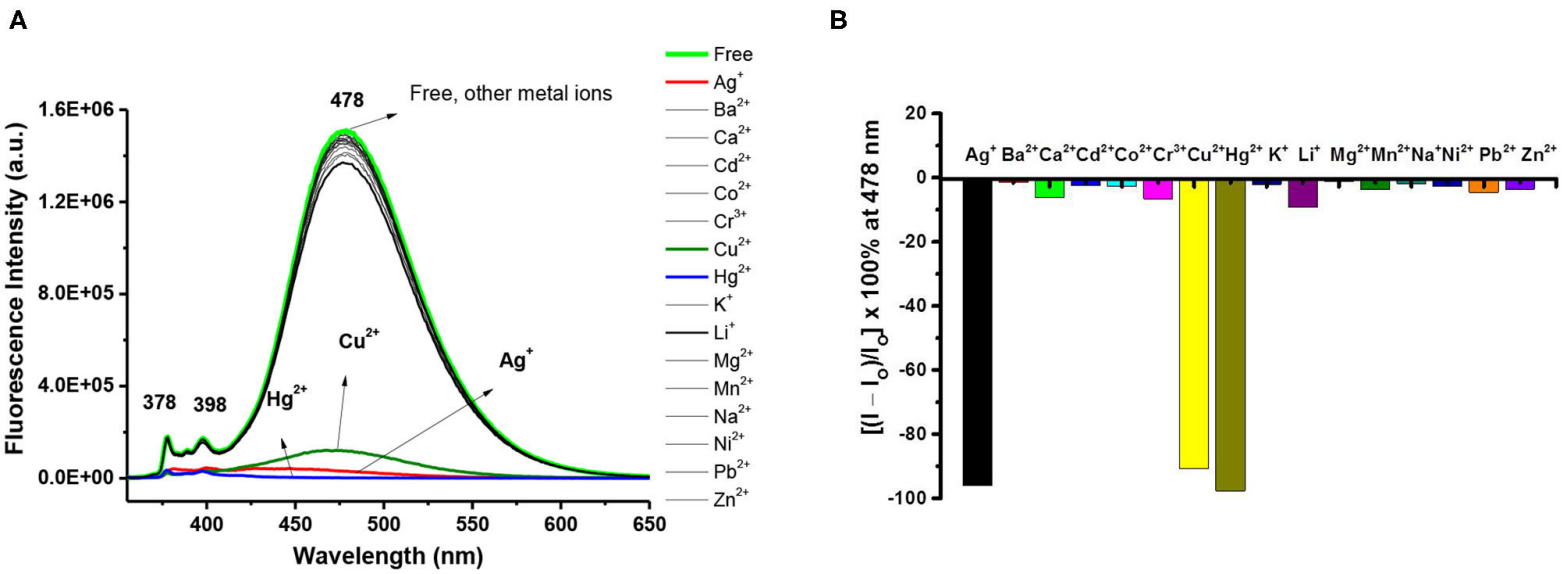
**(A)** Fluorescence changes of ligand **3** (10 μM) by the addition of 10 equiv of various metal perchlorates (Ag^+^, Ba^2+^, Ca^2+^, Cd^2+^, Co^2+^, Cr^3+^, Cu^2+^, Hg^2+^, Li^+^, Na^+^, K^+^, Mg^2+^, Ni^2+^, Pb^2+^, and Zn^2+^) in CHCl_3_/MeOH (v/v, 3:1). Extinction wavelength was 345 nm. **(B)** Percentage fluorescence intensity changes of ligand **3** by the addition of 10 equiv of various metal perchlorates in CHCl_3_/MeOH (v/v, 3:1).

The selectivity of pyrazolylmethylpyrene functionalized calix[4]arenes **3** and **8** toward 15 different perchlorate salts of metal ions (Ag^+^, Ba^2+^, Ca^2+^, Cd^2+^, Co^2+^, Cr^3+^, Cu^2+^, Hg^2+^, Li^+^, Na^+^, K^+^, Mg^2+^, Ni^2+^, Pb^2+^, and Zn^2+^) was then examined by UV-Vis and fluorescence spectroscopy (Supplementary Figures 7–9, 14–16, ESI). The UV-Vis spectra of ligand **3** exhibited small bathochromic shift with significant hypochromic effect when it complexed with Hg^2+^, Ag^+^, and Cu^2+^ ions, but no obvious change was observed for other metal ions. When excited at 345 nm, free ligand **3** (10 μM) in CHCl_3_/MeOH (v/v, 3:1) showed a strong intramolecular excimer emission band around 478 nm and 2 weak monomer emission bands at 378 and 398 nm (Winnik, 1993; Yang et al., 2001; Kim et al., 2003, 2007; Yuasa et al., 2004; Shiraishi et al., 2006; Hung et al., 2009a,b). Both the monomer and excimer emissions of ligand **3** were strongly quenched (>90%) in the presence of 10 equiv of Hg^2+^, Ag^+^, or Cu^2+^ ions (see Figure 1A). The selectivity of ligand **3** toward Cu^2+^ was not further studied because the free phenol groups of calix[4]arene have been previously demonstrated by us (Chang et al., 2008; Senthilvelan et al., 2009) to be readily oxidized by Cu^2+^, which then formed strong complex with the reduced Cu^+^.

In the titration of ligand **3** with Hg^2+^ (or Ag^+^), a small bathochromic shift (λ_max_ from 345 to 346 or 348 nm) was observed in its UV-Vis spectra as the concentration of Hg^2+^ (or Ag^+^) increased; furthermore, several isosbestic points were observed (Supplementary Figures 8a, 9a, ESI) indicating that the free and complexed forms of ligand **3** coexisted in the system. Assuming a 1:1 complexation, the binding constant of ligand **3** with Hg^2+^ and Ag^+^ could be obtained from the Benesi-Hildebrand plot (Benesi and Hildebrand, 1949) of UV-Vis titration experiments to be 2.99 × 10^3^ and 3.83 × 10^3^ M^−1^, respectively (Supplementary Figures 7b, 9f, ESI).

Both the monomer and excimer emissions of ligand **3** were drastically quenched upon adding 0.2 to 1.0 equiv of Hg^2+^ (or Ag^+^), and the fluorescence emission reached low (or very low) intensity values after adding 2 equiv of Hg^2+^ (or Ag^+^). By using the initial slope of the linear Stern–Volmer plots, the fluorescence quenching constant of ligand **3** with Hg^2+^ and Ag^+^ was calculated to be 2.99 (±0.11) × 10^4^ and 3.00 (±0.26) × 10^4^ M^−1^, respectively (Supplementary Figures 8c, 9c, ESI). In principle, there are two types of excimer for pyrene compounds: dynamic and static (Winnik, 1993; Yang et al., 2001; Hung et al., 2014); the former results from a pyrene dimer formed in the excited state, whereas the latter arises from a pyrene dimer that exists in the ground state. The upward-curving Stern-Volmer plots indicate that the fluorophore can be quenched both by collisions with the excited states and by complexation with the quencher in the ground state (Lakowicz, 1999). Consequently, the fluorescence quenching of ligand **3** with Hg^2+^ and Ag^+^ may attribute to dynamic quenching, static quenching, and aggregation (Ebeid et al., 1988; Lakowicz, 1999; Luo et al., 1999). Job plot experiments of ligand **3** with Hg^2+^ showed that the excimer emissions of ligand **3** reached a maximum at 0.5 molar fractions (Supplementary Figure 8d, ESI), indicating a 1:1 complexation mode of compound **3** with Hg^2+^. Interestingly, the Job plot of ligand **3** with Ag^+^ showed a maximum at 0.4 molar fraction of ligand **3** (Supplementary Figure 9d, ESI), indicating a 2:3 complexation mode of ligand **3** toward Ag^+^ ion. High-resolution mass spectrometry proved that a 1:1 complex of **3**•Ag^+^ exists (Supplementary Figure 9e, ESI); however, we did not observe the mass peaks of **3**•(Ag^+^)_2_.

To gain insight into the possible binding modes and binding ratios of ligand **3** with Hg^2+^ or Ag^+^, we further carried out the ^1^H NMR titration experiments of ligand **3** with Hg^2+^ and Ag^+^. The methylene bridge protons of free ligand **3** exhibiting **two** doublets at 4.51 and 3.51 ppm (Δδ = +1.00) were upfield shifted to 3.18 and 3.04 ppm (Δδ = +0.14) when 1 equiv of Ag^+^ was added (Supplementary Figure 10, ESI), *implying that the cone conformation of calix[4]arene in free ligand* ***3****was distorted to pinched cone conformation* (Scheerder et al., 1996) *when complexed with Ag*^+^. The pyrazole methine protons, H-b of ligand **3**, were strongly downfield shifted from 6.95 to 6.93 ppm (overlapped with aromatic signals of calix[4]arene) to 8.2 ppm after they were complexed with Ag^+^ ion; however, the other pyrazole methine protons, H-c, were slightly upfield shifted by 0.08 ppm (Supplementary Figure 10, ESI). The chemical shifts of pyrazole protons H-b and H-c of ligand **3** were confirmed by their mutual correlation in H,H-COZY spectrum (Supplementary Figure 11, ESI). Furthermore, the methylene protons H-d (5.27 ppm), linking the pyrazole and calix[4]arene units, were upfield shifted to 3.95 ppm, whereas the methylene protons H-a (5.95 ppm), linking the pyrazole and pyrene units, were downfield shifted by 0.20 ppm (Supplementary Figures 10, 12, ESI). Interestingly, all the proton signals of ligand **3** were broadened in the presence of 0.25 to 0.75 equiv of Ag^+^ but became sharp again after adding 1 equiv of Ag^+^. Such a peak broadening may be attributed to the faster rate of the complexation-decomplexation of the ligand **3** with Ag^+^ than the NMR time scale at 25°C (Ikeda and Shinkai, 1994). Moreover, it implies that the binding ratio of ligand **3** with Ag^+^ was 1:1. In contrast, the complexation of ligand **3** with Hg^2+^ showed that all proton signals were sharp and new sets of methylene bridge protons were generated (3.91, 3.31, 2.89, and 2.78 ppm) when Hg^2+^ (0.25 to 2 equiv) was added, which indicated the slower rate of complexation-decomplexation of the ligand **3** with Hg^2+^. When ligand **3** was complexed with Hg^2+^, the chemical shifts of H-a, H-b, H-c, and H-d were downfield shifted by 0.40, 1.88, 0.04, and 0.18 ppm, respectively (Supplementary Figure 13, ESI).

**Figure 2:**
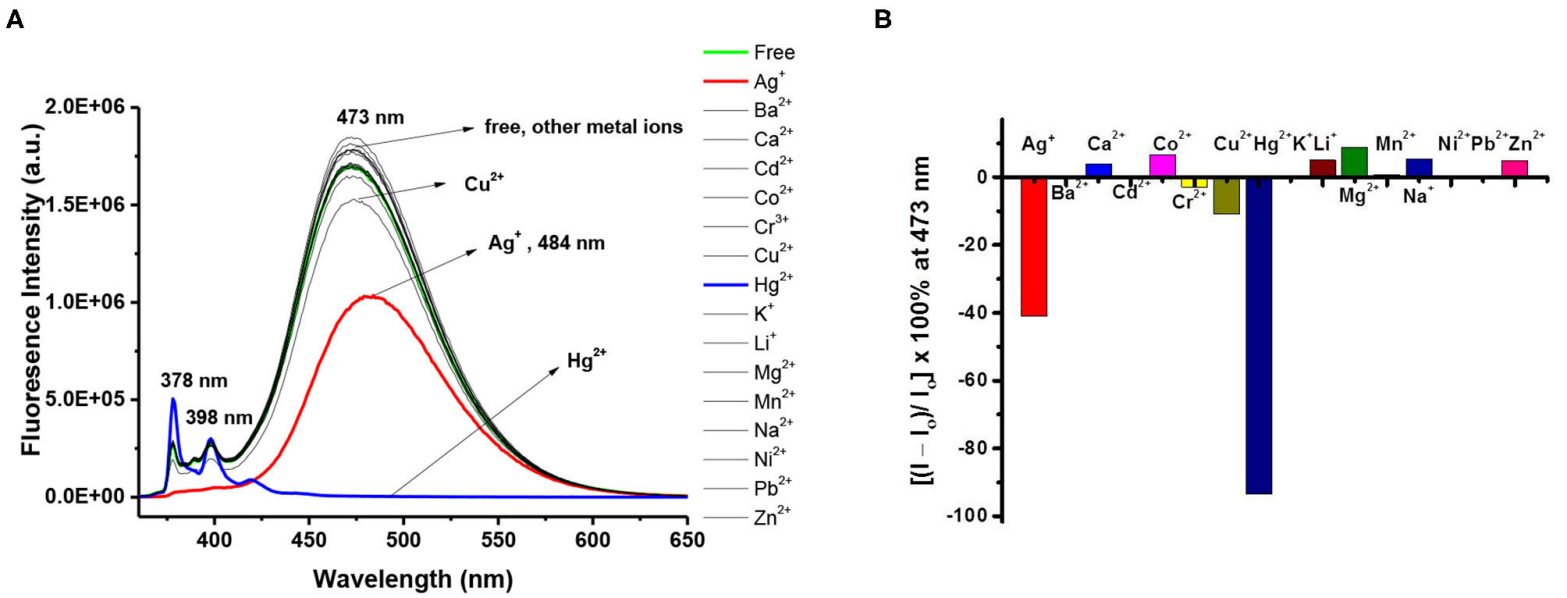
**(A)** Fluorescence changes of ligand **8** (10 μM) by the addition of 10 equiv of various metal perchlorates (Ag^+^, Ba^2+^, Ca^2+^, Cd^2+^, Co^2+^, Cr^3+^, Cu^2+^, Hg^2+^, Li^+^, Na^+^, K^+^, Mg^2+^, Ni^2+^, Pb^2+^, and Zn^2+^) in CHCl_3_/MeOH (v/v, 3:1). Excitation wavelength was 347 nm. **(B)** Percentage fluorescence intensity changes of ligand **8** by the addition of 10 equiv of various metal perchlorates in CHCl_3_/MeOH (v/v, 3:1).

To further study the ion selectivity of the 1,3-alternate calix[4]arene **8**, the binding properties of ligand **8** (10 μM) in CHCl_3_/MeOH (v/v, 3:1) toward 15 different metal ions (10 equiv) were assessed by UV-Vis (Supplementary Figure 14, ESI) and fluorescence spectroscopy (*vide infra*). The absorbance of UV-Vis spectra of **8** decreased when Ag^+^ or Hg^2+^ was added, and it showed a small bathochromic shift (from 345 to 349 nm) for Ag^+^; however, the other metal ions did not show any significant change in λ_max_. Among the 15 metal ions screened, the fluorescence intensity of ligand **8** was severely quenched by Hg^2+^, to a much less extent by Ag^+^ ions, and was little or not affected by Cu^2+^ and other metal ions (Figure 2A). The results differ with those of ligand **3** (cf. Figure 2B with Figure 1B) implying that the lower-rim bis-pyrazole units in ligand **8** alone cannot bind Cu^2+^; in order for ligand **8** to bind Cu^2+^, it needs further assistance from the two franking phenols as deployed in ligand **3** and is consistent with our previous observations that free phenols of the calix[4]arene can be readily oxidized by Cu^2+^ to calixquinones and then complexed with the reduced Cu^+^ (Chang et al., 2008; Senthilvelan et al., 2009). Upon the addition of Hg^2+^ to ligand **8**, the intensity of the excimer emission of ligand **8** decreased by 93%, while a concomitant enhancement on one of its monomer emission bands (λ_max_ 378 nm) appeared. In contrast, both monomer and excimer emissions of ligand **8** were quenched by Ag^+^ to about 41% of its original intensity, and the excimer emission maximum was red shifted by 11 nm.

In the UV-Vis titration experiments of ligand **8** with Hg^2+^ and Ag^+^, the absorbance gradually decreased with slight red shift as the concentration of Hg^2+^ or Ag^+^ increased, and several isosbestic points were observed indicating that free and complexed forms of ligand **8** with these two metal ions coexisted in the system. The spectral features of the fluorescence titration of ligand **8** with Ag^+^ showed that both the monomer and excimer emissions of the appended pyrenes of ligand **8** were moderately quenched upon adding 0.2 to 2.0 equiv of Ag^+^ and reached a low value at 2 equiv of Ag^+^; however, titration of ligand **8** with Hg^2+^ showed a severe quenching on the excimer emission but an enhancement on the monomer emission at 378 nm (Supplementary Figures 15, 16, ESI). Hill plot analysis for 1:2 ligand-to-metal complexation was considered, and to our delight, the stepwise complexation constants of ligand **8** with Hg^2+^ and Ag^+^ were obtained with an excellent fitting of the experimental data to Equation (1): (Cielen et al., 1998)

(1) $$\begin{array}{r} log\left[\left(I-I_{\text{min}}\right)/\left(I_{\text{o}}-I\right)\right]=n\times p\left[M\right]-logK_{\text{s}} \end{array}$$

where [*M*] is the concentration of the free metal ion, *n* is the mole ratio of metal ion to ligand, and *I*_o_ and *I*_min_ are the fluorescence intensities of the free ligand **8** and of the complex at saturated level, respectively. *K*_s_ is the association constant of ligand **8** with metal ion. A plot of log [(*I* – *I*_min_)/(*I*_o_ – *I*)] vs. *p*[*M*] yields a straight line with an intercept of –log *K*_s_ and a slope of Hill coefficient, *n*. Consequently, the Hill coefficient of ligand **8** toward Hg^2+^ and Ag^+^ was determined to be 2.54 and 2.42, respectively, indicating that the interaction coefficient was around 2 in both cases, and the association constant of ligand **8** with Hg^2+^ and Ag^+^ was determined to be 1.46 × 10^12^ and 9.24 × 10^11^ M^−2^, respectively (Weiss, 1997; Takeuchi et al., 1998). Assuming that the binding constant of first equivalent of Hg^2+^ (or Ag^+^) by ligand **8** is similar to that by ligand **3** (2.99~3.83 × 10^3^ M^−1^), the binding constant of second equivalent of Hg^2+^ (or Ag^+^) would then be close to 2.41~4.88 × 10^8^ M^−1^ based on the Hill plots. However, there is an increasing debate regarding the interpretation of the Hill coefficient (Weiss, 1997; Thordarson, 2011); thus, the Hill coefficient is best thought of as an “interaction” coefficient, reflecting the extent of cooperativity among multiple ligand sites. The Job plot experiments of ligand **8** with Hg^2+^ and Ag^+^ were carried out by using fluorescence intensity of the emission maxima as a function of the mole fraction of respective metal ions, and the binding ratio of 1:2 was also obtained for both Hg^2+^ and Ag^+^ (Supplementary Figures 15c,d, 16c,d, ESI). Moreover, the ESI-MS data of the complex **8**•(Ag^+^)_2_•${\text{ClO}}_{4}^{-}$ provided further evidence for the 1:2 ligand-metal complex (Supplementary Figure 18, ESI).

**Figure 3:**
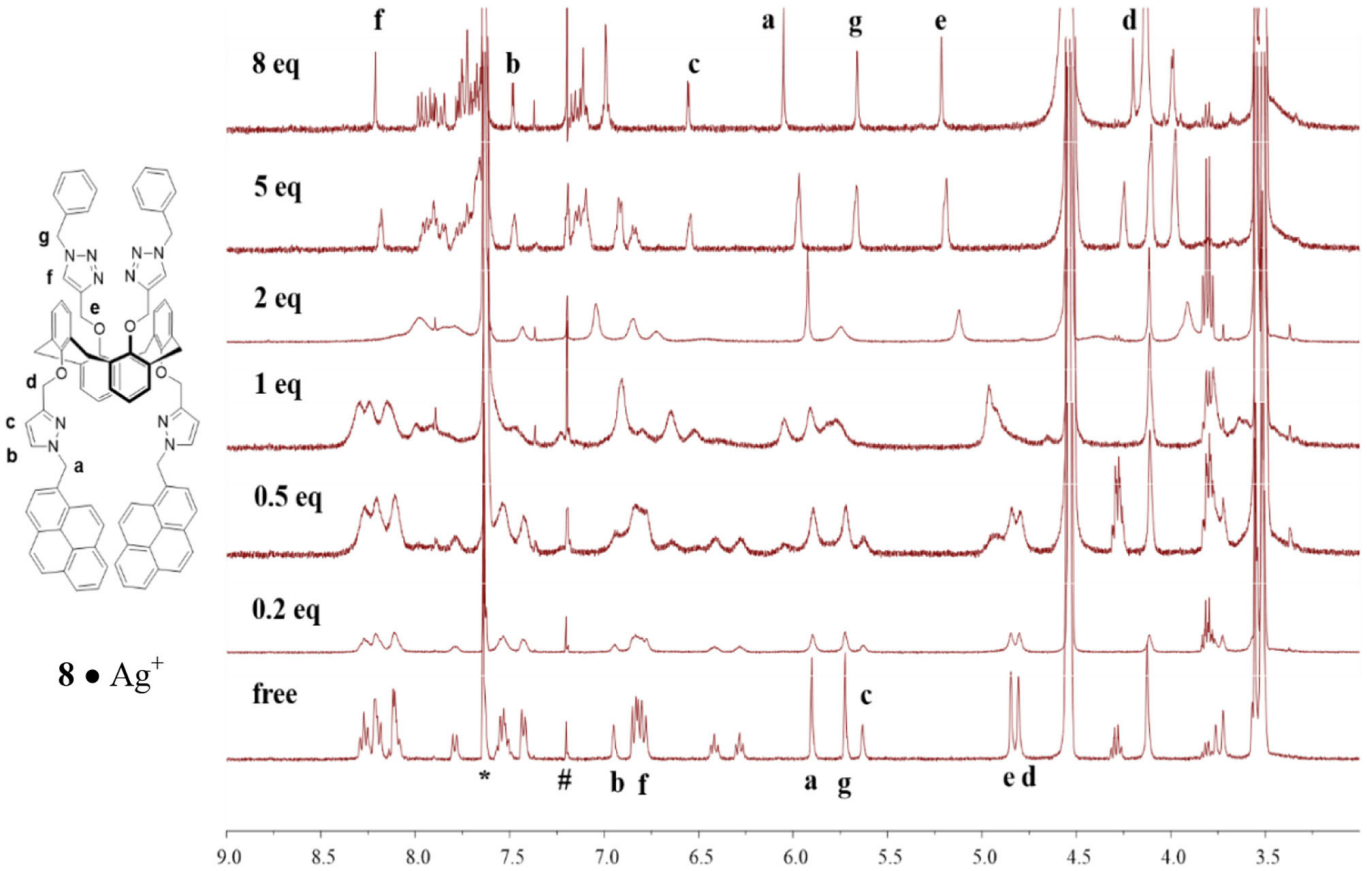
^1^H NMR titration spectra of ligand **8** (1.33 mM) in the presence of different amount of AgClO_4_ in CDCl_3_/CD_3_OH (v/v, 3:1); where * denotes signals from residual CHCl_3_ and # denotes signals from external CHCl_3_.

To obtain structural information and conformational change in the complexes formed between ligand **8** and Ag^+^ (or Hg^2+^), we carried out ^1^H NMR titration experiments on ligand **8** (1.33 mM) with different equiv of Ag^+^ (or Hg^2+^) in CDCl_3_/CD_3_OH (v/v, 3:1) co-solvent. Upon adding 0.2 to 2 equiv of Ag^+^ to ligand **8**, most of the proton signals were broadened, which could be attributed to the metal-tunneling effect between the two cation-binding sites in the 1,3-alternate conformation of ligand **8** (Ikeda and Shinkai, 1994; Ikeda et al., 1997; Ho et al., 2011). All proton signals became sharp again after adding 5 equiv of Ag^+^ to the solution of ligand **8** (see Figure 3). Variable temperature ^1^H NMR spectra of ligand **8** with 1 equiv of Ag^+^ were collected to interpret the metal-tunneling motions. At −50°C, the proton signals of the **8**•Ag^+^ complex appeared quite sharp and well-separated from each other compared with those of the free ligand **8** at room temperature (Supplementary Figure 17, ESI) (Ikeda et al., 1997; Ho et al., 2011).

Upon titration of ligand **8** with 8 equiv of Ag^+^, the methylene protons H-a and pyrazole protons H-b and H-c were downfield shifted by 0.15, 0.53, and 0.92 ppm, respectively; whereas the methylene protons H-d (4.70 ppm), linking the pyrazole and calix[4]arene units, were upfield shifted by 0.60 ppm. Moreover, the proton H-f of triazole at 6.78 ppm was downfield shifted to 8.21 ppm (+1.43 ppm), implying the very important role of the triazole as a ligand in coordinating Ag^+^ ions. The methylene protons H-e (4.85 ppm), linking the triazole and the calix[4]arene units, were downfield shifted by 0.36 ppm, and the other methylene protons H-g (5.72 ppm), linking the triazole and the phenyl units, were upfield shifted by 0.06 ppm. One can see that all proton signals were broadened when 0.2 to 2 equiv of Ag^+^ was added, which was consistent with the metal ion tunneling between the upper-rim bis-triazolylmethylphenyl binding site and the lower-rim bis-pyrazolyl-methylpyrene binding site (Ikeda and Shinkai, 1994; Ikeda et al., 1997; Shinkai, 2001; Ho et al., 2011). ^1^H NMR spectra of ligand **8** (1.33 mM) in the presence of various equiv of Hg^2+^ were also carried out (Supplementary Figure 19, ESI); however, precipitation occurred when 2 equiv of Hg^2+^ was added to the solution of ligand **8**. Proton signals of the lower rim (H-a, H-b, H-c, and H-d) were slightly downfield shifted when 0.2 to 1.5 equiv of Hg^2+^ was added. Furthermore, a new set of signals was observed, which could be due to the slow exchange rate of ligand **8** with Hg^2+^. Based on all observations stated above, we proposed that ligand **3** forms a 1:1 complex with Hg^2+^ (or Ag^+^) using the lower-rim bis-pyrazolylmethylpyrenes as the binding site. In contrast, ligand **8** forms a strong 1:2 complex with Hg^2+^ (or Ag^+^) using the two cavities, which consisted of the bis-pyrazolylmethylpyrenes on the one rim and bis-triazolylmethylphenyls on the other rim, of the 1,3-alternate conformation of ligand **8**.

## Conclusion

**Scheme 2:**
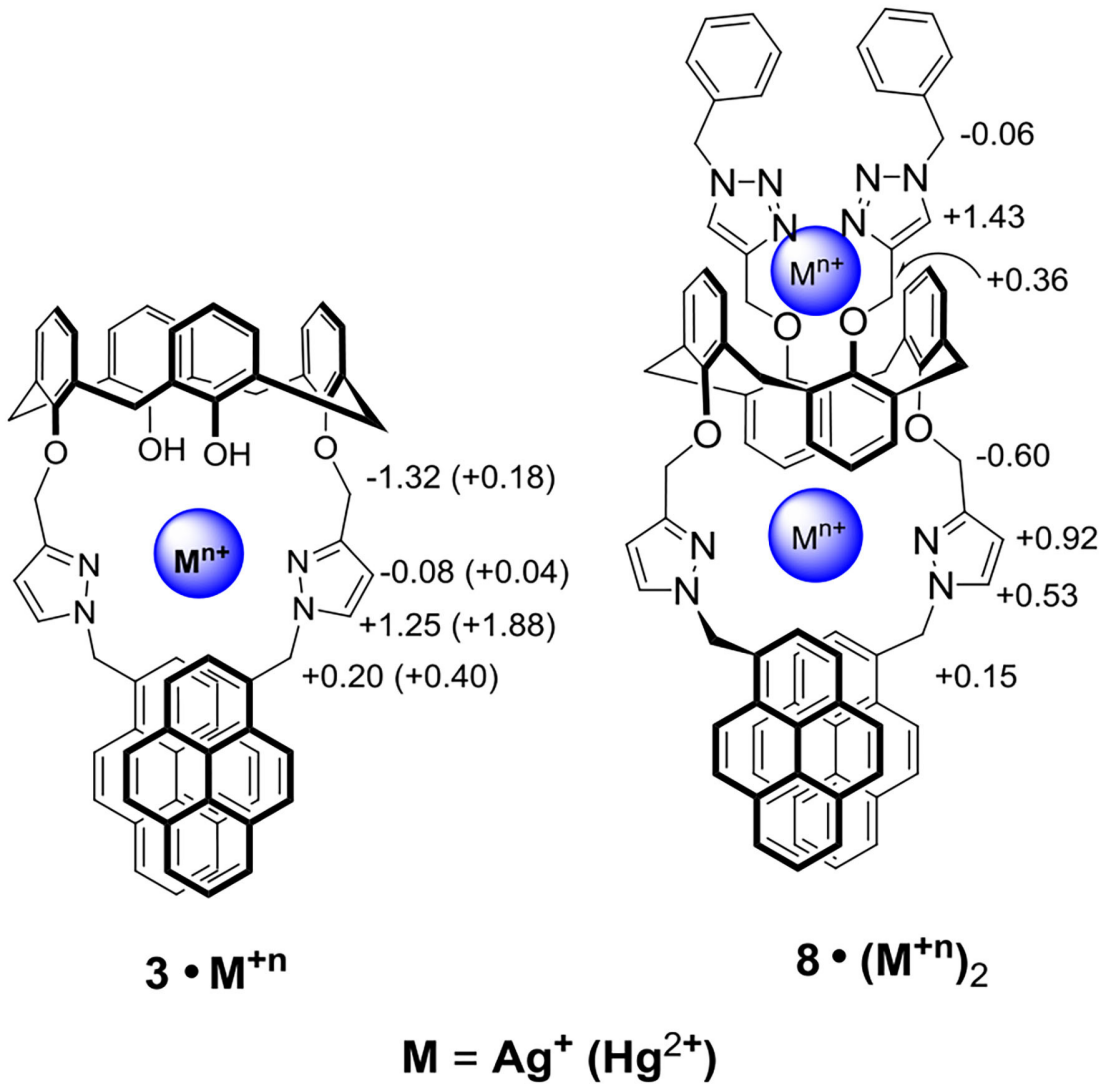
Two possible binding modes of ligands **3** and **8** with Ag^+^ and Hg^2+^ ions, where the chemical shift differences (Δδ = δ_complex_ – δ_ligand_) are shown for Ag^+^ (data for Hg^+2^ complexes are in parentheses).

We report here the synthesis of a 1,3-alternate calix[4]arene **8** with bis-pyrazolyl-methylpyrenes on the one rim and bis-triazolylmethylphenyl on the other rim as a homoditropic fluorescent sensors for Hg^2+^ and Ag^+^ ions. Calix[4]arene **3**, with lower-rim bispyrazolylmethylpyrenes in cone conformation, was also synthesized as a control compound. UV-Vis and fluorescence spectra were used for metal ions screening, and we found that free ligands **3** and **8** in CHCl_3_/MeOH (v/v, 3:1) exhibited strong excimer emissions; however, they both showed high selectivity toward Hg^2+^ and Ag^+^ ions with strong fluorescent quenching. Job plot and ^1^H-NMR titration experiments supported that ligand **3** formed 1:1 complexes with Hg^2+^ but it formed a 2:3 complex with Ag^+^; however, ligand **8** formed strong 1:2 complexes with Hg^2+^ and Ag^+^ (Scheme 2). The binding constant of ligand **3** with Hg^2+^ and Ag^+^ was determined by Benesi-Hilderbrand plots of the UV-vis titration experiments to be 2.99 × 10^3^ and 3.83 × 10^3^ M^−1^, respectively, while the homoditropic sensing of ligand **8** with two Hg^2+^ or two Ag^+^ ions was determined by Hill plots to be 1.46 × 10^12^ and 9.24 × 10^11^ M^−2^, respectively. Ligand **8** forms a strong complex with two Hg^2+^ (or Ag^+^) ions using the bis-pyrazolylmethylpyrenes on the one rim and bis-triazolylmethylphenyls on the other rim of the 1,3-alternate conformation, which represents one of the few highly selective fluorescent sensors for the homoditropic sensing of Hg^2+^ and Ag^+^ ions.

## Data Availability Statement

All datasets generated for this study are included in the article/Supplementary Material.

## Author Contributions

Y-JC and W-SC designed the work and wrote the manuscript. Y-JC, M-YC, K-TL, H-CH, and H-CN carried out the experiments. K-TL and L-CS performed the spectroscopic experiments. W-SC revised and edited the manuscript. All authors reviewed the manuscript and have agreed to its publication.

## Conflict of Interest

The authors declare that the research was conducted in the absence of any commercial or financial relationships that could be construed as a potential conflict of interest.
