# Supplementary material for: 1,3-Alternate Calix[4]arene Functionalized With Pyrazole and Triazole Ligands as a Highly Selective Fluorescent Sensor for Hg2+ and Ag+ Ions
Source: Front Chem. 2020 Nov 12;8:593261. doi: 10.3389/fchem.2020.593261 (PMC7688584; doi:10.3389/fchem.2020.593261)
Supplement: Supplementary file 1 [file Data_Sheet_1.pdf]

Supplementary Material

Page	Table of Contents
S2-S3	Experimental detail for the synthesis of compound **2**.
S4	**Figures S1** and **S2.** ^{1}H and DEPT spectra of ligand **3** in $CDCl_3$.
S5	**Figures S3** and **S4.** ^{1}H and ^{13}C NMR spectra of compound **5** in $CDCl_{3.}$
S6	**Figures S5** and **S6.** ^{1}H and ^{13}C NMR spectra of ligand **8** in $CDCl_{3.}$
S7	**Figure S7** UV-Vis spectra of compound **3** with 10 equiv of various metal perchlorates and **Figure S8.** UV-vis and fluorescence titration studies of ligand
S8	**Figure S9**. UV-vis and fluorescence titration studies of ligand **3** with Ag^{+}.
S9	**Figure S10.** ^{1}H NMR titration of **3** in $CDCl_3/CD_3OH$ (v/v, 3:1) in the presence of different equiv of $AgClO_4$ and **Figure S11.** H,H-COSY of **3** in the presence
S10	**Figure S12.** 2D NOESY of **3** in the presence of 2 equiv of Ag^{+} and **Figure S13.** ^{1}H NMR titration of **3** in $CDCl_3/CD_3OH$ (v/v, 3:1) in the presence of different
S11	**Figure S14.** UV-Vis spectra of ligand **8** with 10 equiv of various metal perchlorates.
S12	**Figures. S15.** UV-Vis, fluorescence spectra, Hill plot, and Job plot of ligand **8** with Ag^{+}.
S13	**Figures. S16.** UV-Vis, fluorescence spectra, Hill plot, and Job plot of ligand **8** with Hg^{2+}. .
S14	**Figure S17.** Variable temperature ^{1}H NMR (-50 to 50 ^{o}C) spectra of **8** with 1 equiv of $AgClO_4$ in $CDCl_3/CD_3OH$ (v/v, 3:1).
S15	**Figure S18.** The ESI-MS data of the complex **8•**$(Ag^{+})_2$.
S16	**Figure S19.** The ^{1}H NMR titration spectra of ligand **8** in the presence of different equiv of $Hg(ClO_4)_2$ in $CDCl_3/CD_3OH$ (v/v, 3:1).

Experimental detail for the synthesis of compound 2.

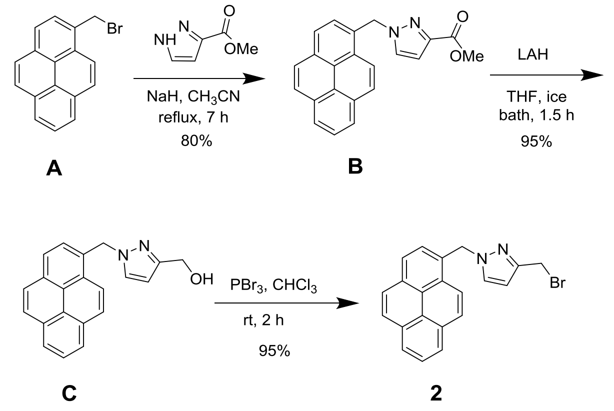

Scheme S1

Synthesis of compound B. Compound **A**[S1] was prepared according to literature procedures, which was then used for the synthesis of compound **B**. A mixture of methyl 1*H*-pyrazole-3-carboxylate 0.27 g (2.11 mmol) and sodium hydride 60% dispersion 105 mg (2.54 mmol) in 75 mL CH_3CN was stirred vigorously at 0 ^oC for 15 min. The solution was added 1-(bromomethyl)pyrene **A** 0.50 g (1.69 mmol) and was heated to reflux for 7 h. The solvent was removed by reduced pressure. The solution was extracted with ethyl acetate/water (v/v, 1.5/1) and dried over anhydrous $MgSO_4$. The residue was obtained after evaporation of the solvent and was subjected to a silica gel column chromatography (ethyl acetate/*n*-hexane = 1/3) to afford a white yellow solid (0.35 mg, 80 %); R_f = 0.2 (ethyl acetate/*n*-hexane = 1/3), mp 112–114 ^oC. ^1H NMR ($CDCl_3$, 400 MHz) δ_H 8.24–8.01 (m, 8H), 7.83 (d, J = 7.8 Hz, 1H), 7.08 (d, J = 2.4 Hz, 1H), 6.75 (d, J = 2.4 Hz, 1H), 3.07 (m, 3H); ^{13}C NMR ($CDCl_3$, 75.4 MHz) δ_C 163.3 (Cq), 143.6 (Cq), 132.4 (Cq), 131.6 (Cq), 131.0 (CH),130.9 (Cq), 129.8 (Cq), 129.3 (CH), 128.6 (CH), 128.1 (CH), 127.8 (Cq), 127.7 (CH), 126.7 (CH), 126.2 (CH), 126.1 (CH), 125.4 (Cq), 125.3 (CH), 124.9 (Cq), 122.5 (CH), 109.8 (CH), 55.6 (CH_2), 52.5 (CH_3). LREI-MS: *m/z* 215.1 [M - 125], 340.1 $[M]^+$; HRMS: *m/z* calcd for $C_{22}H_{16}N_2O_2$ 340.1212, found 340.1202.

Synthesis of compound C from compound B. A mixture of methyl 1-((pyren-3-yl)methyl)- 1*H*-pyrazole-3-carboxylate (**B**) 1.00 g (2.94 mmol) and lithium aluminum hydride 0.13 mg (3.67 mmol) in 250 mL dried THF was stirred at 0 ^oC under nitrogen for 2 h. The reaction was ceased by adding 1 mL of 1N NaOH (aq) at 0 ^oC. The mixture was filtrated and extracted (150 mL × 3) in a mixture of ethyl acetate/water (1.5/1). The organic layer was rinsed with water thrice and dried over anhydrous $MgSO_4$. The solvent of the organic layer was removed under reduced pressure to give a yellow solid (0.90 g, 97 %); R_f = 0.08 (EtOAc/*n*-hexane = 1/3). Mp 128–130 ^oC. ^1H NMR ($CDCl_3$, 400 MHz) δ_H 8.26–7.97 (m, 8H), 7.83 (d, J = 7.8 Hz, 1H), 7.16 (d, J = 2.3 Hz, 1H), 6.21 (d, J = 2.3 Hz, 1H), 6.01 (s, 2H), 4.75 (d, J = 5.9 Hz, 2H). ^{13}C NMR ($CDCl_3$, 100 MHz) δ_C 152.2 (Cq), 131.6 (Cq), 131.2 (Cq), 130.6 (Cq), 130.3, (CH), 129.1 (Cq), 128.6 (Cq),

128.5 (CH), 127.8 (CH), 127.2 (CH), 127.2 (CH), 126.2 (CH), 125.6 (CH), 125.5 (CH), 124.9 (Cq), 124.8 (CH), 124.5 (Cq), 122.3 (CH), 104.4 (CH), 59.1 (CH_2), 54.0 (CH_2).

Synthesis of compound 2 from compound C. To a solution of compound **C** 1.00 g (3.20 mmol) in 60 mL of $CHCl_3$ was added dropwise 0.15 mL (1.12 mmol) of phosphorous tribromide and the resulting solution was refluxed for 2 h. The mixture was cooled and extracted thrice with CH_2Cl_2/water (1.5/1). The ether layer was dried over anhydrous $MgSO_4$ and the solvent was removed under reduced pressure to give a yellow solid (1.14 g. 95 %); R_f = 0.45 (ethyl acetate/*n*-hexane = 1/3). ^1H NMR ($CDCl_3$, 400 MHz) δ_H 8.21–8.00 (m, 8H), 7.81 (d, *J* = 7.8 Hz, 1H), 7.11 (d, *J* = 2.1 Hz, 1H), 6.26 (d, *J* = 2.1 Hz, 1H), 5.98 (s, 2H), 4.57 (s, 1H). ^{13}C NMR ($CDCl_3$, 100 MHz) δ_C 148.8 (Cq), 131.7 (Cq), 131,1 (Cq), 130.6 (CH), 130.5 (Cq), 129.2 (Cq), 128.6 (CH), 128.3 (Cq), 127.9 (Cq), 127.3 (CH), 127.2 (CH), 126.2 (CH), 125.6 (CH), 125.5 (CH), 124.9 (Cq), 124.8 (CH), 124.5 (Cq), 122.2 (CH), 106.1 (CH), 54.3 (CH_2), 25.4 (CH_2). EI-MS: *m*/*z* 215 $(M - 158)^+$, 373 $(M)^+$, 375 $(M + 2)^+$.

Reference

S1. Soto, E., MacDonald, J. C., Cooper, C. G. F., and McGimpsey, W. G. (2003). *J. Am. Chem. Soc.* 125, 2838.

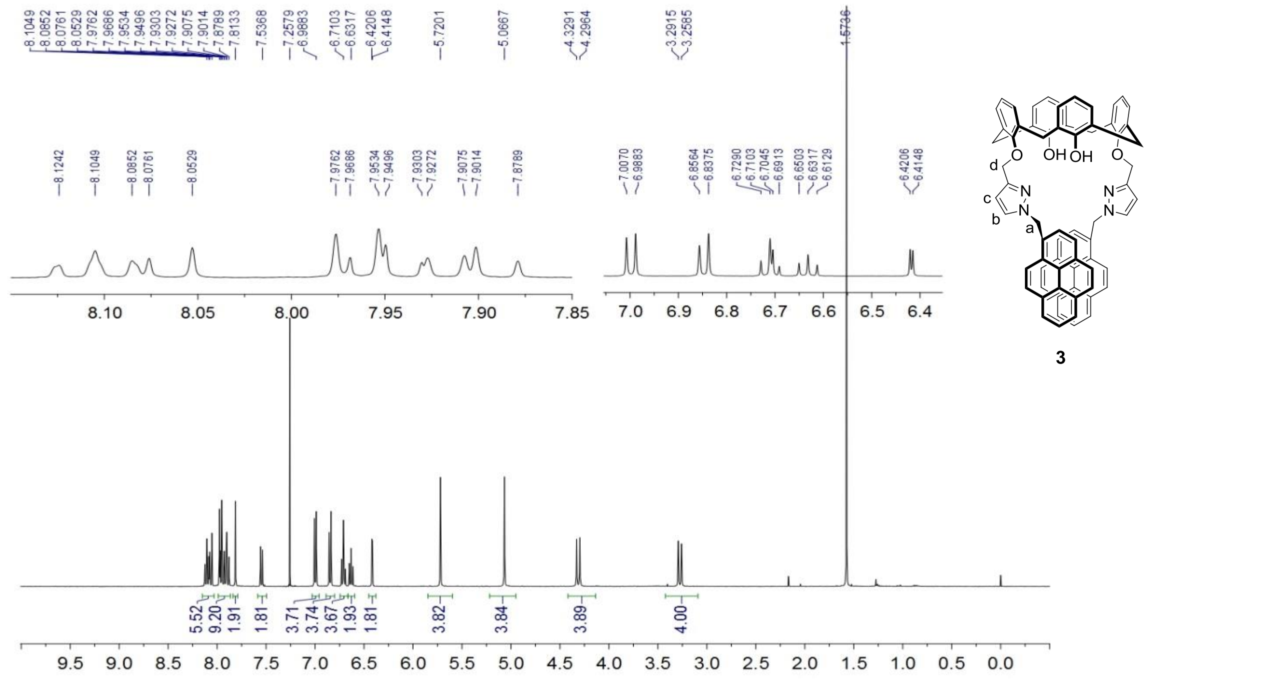

Figure S1. ^{1}H NMR (400 MHz, $CDCl_3$) spectra of 25,27-Bisoxy-(3-methyl-1-pyren-1-ylmethyl-1*H*-pyrazole)-26,28-dihydroxycalix[4]arene **3.**

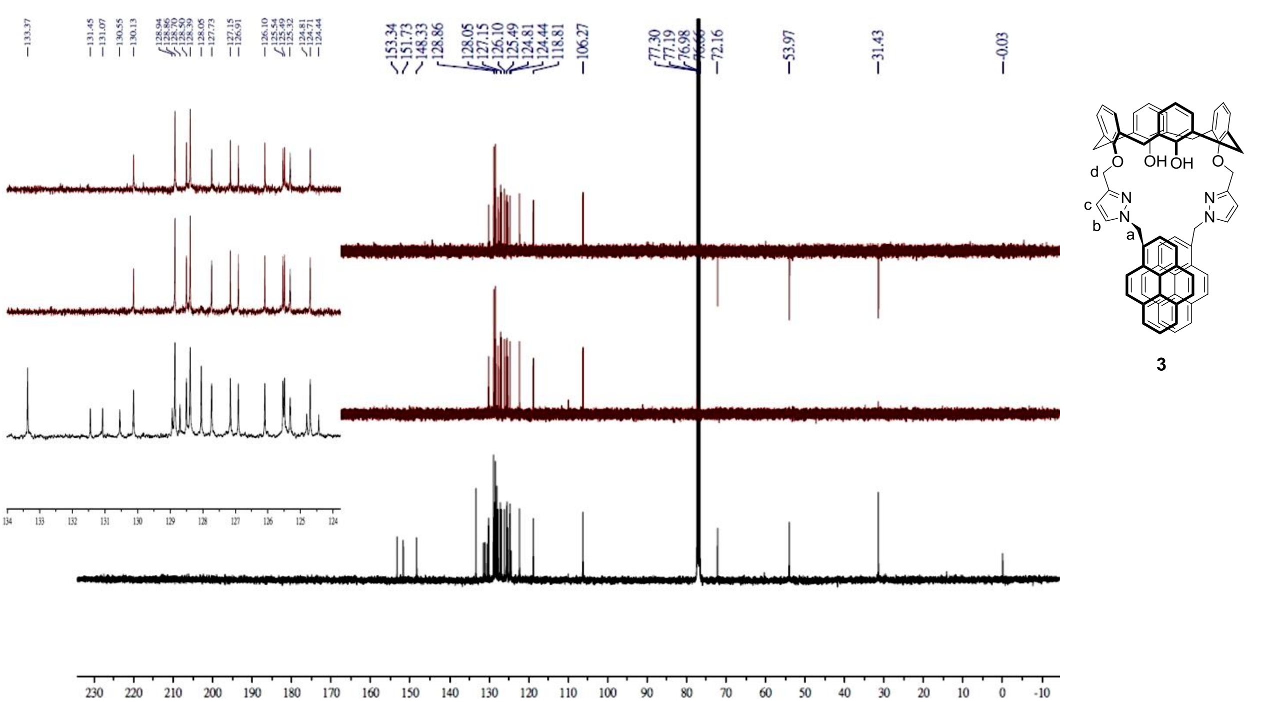

Figure S2. ^{13}C- and DEPT NMR (100 MHz, CDCl3) spectra of 25,27-Bisoxy-(3-methyl-1-pyren-1-ylmethyl-1*H*-pyrazole)-26,28-dihydroxycalix[4]arene **3.**

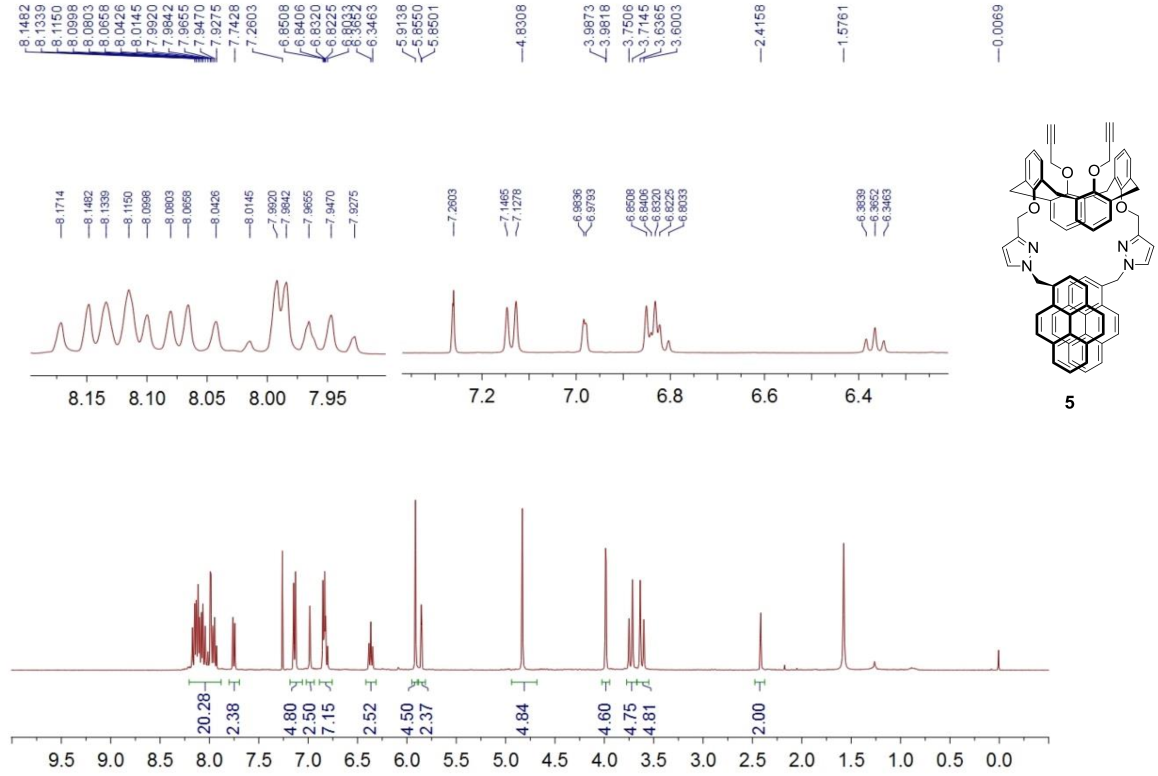

Figure S3. ^{1}H NMR (400 MHz, $CDCl_3$) spectra of 1,3-alternate calix[4]arene **5.**

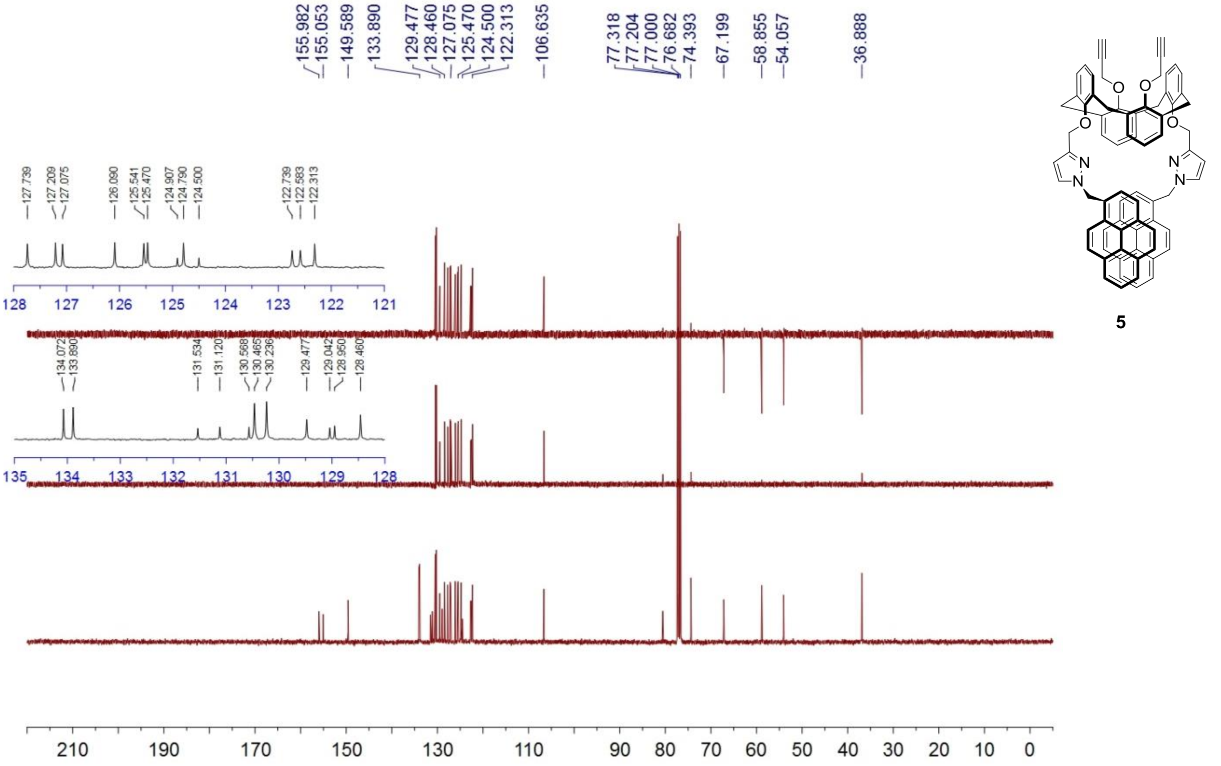

Figure S4. ^{13}C- and DEPT NMR (100 MHz, CDCl3) spectra of 1,3-alternate calix[4]arene **5.**

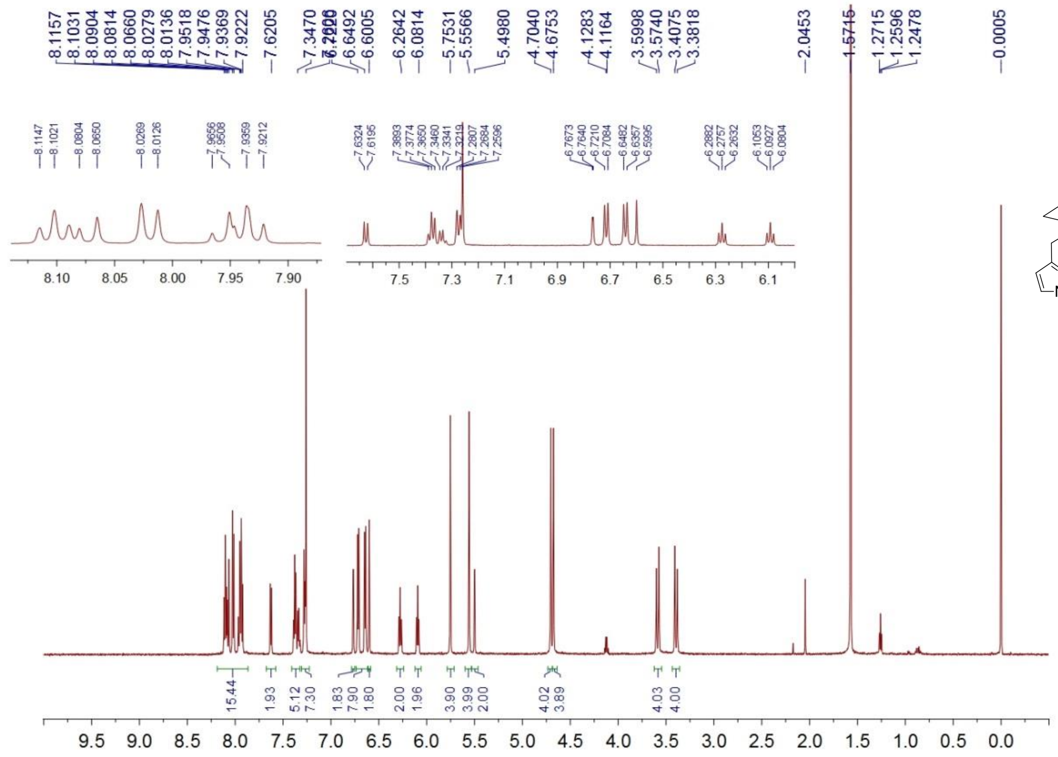

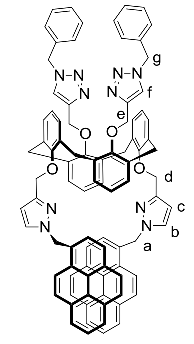

8

Figure S5. ^{1}H NMR (600 MHz, $CDCl_3$) spectra of 1,3-alternate calix[4]arene **8.**

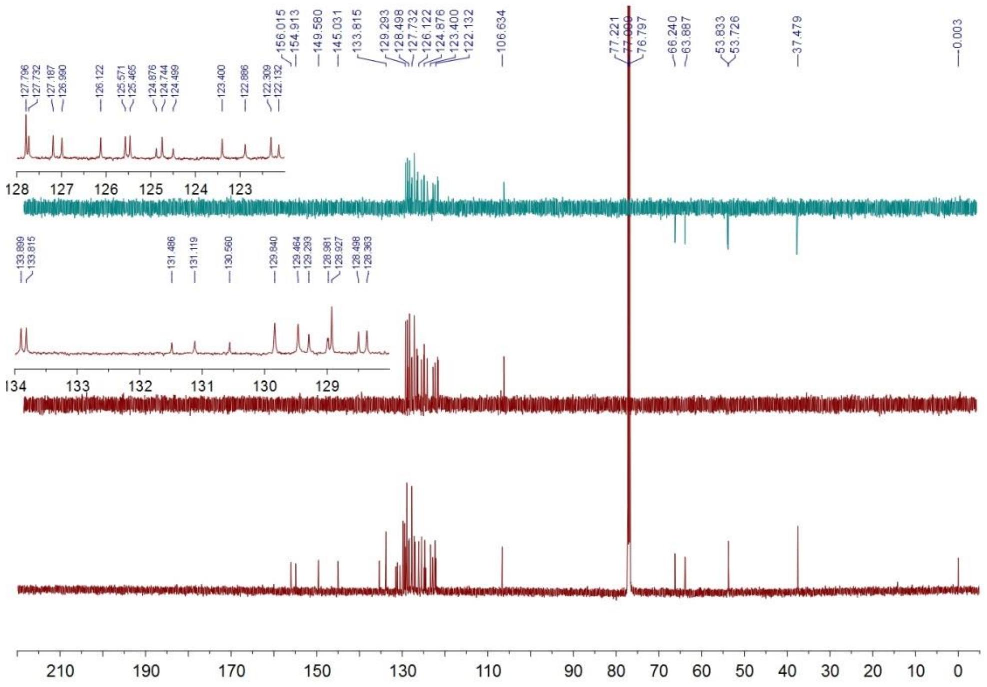

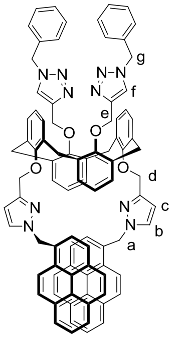

8

Figure S6. ^{13}C- and DEPT NMR (150 MHz, CDCl3) spectra of 1,3-alternate calix[4]arene **8.**

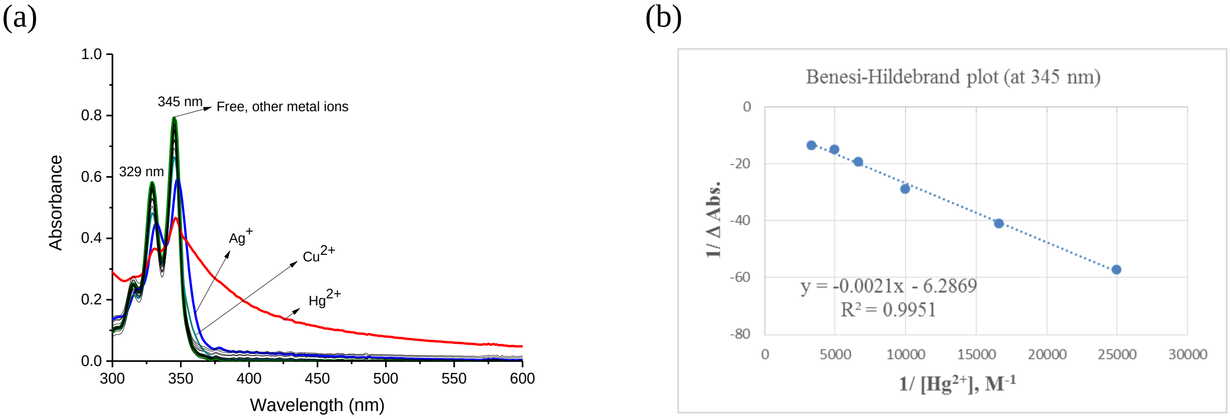

Figure S7. (a) UV-Vis spectra of ligand **3** (10 μM) by the addition of 10 equiv of various metal perchlorates (Ag^{+}, Ba^{2+}, Ca^{2+}, Cd^{2+}, Co^{2+}, Cr^{3+}, Cu^{2+}, Hg^{2+}, Li^{+}, Na^{+}, K^{+}, Mg^{2+}, Ni^{2+}, Pb^{2+}, and Zn^{2+}) in co-solvent $CHCl_3$/MeOH (v/v, 3:1) and (b) Corresponding Benesi-Hildebrand plot of the UV-vis absorption of ligand **3** (10 μM) at 345 nm with various equiv. of $Hg(ClO_4)_2$ at 298K.

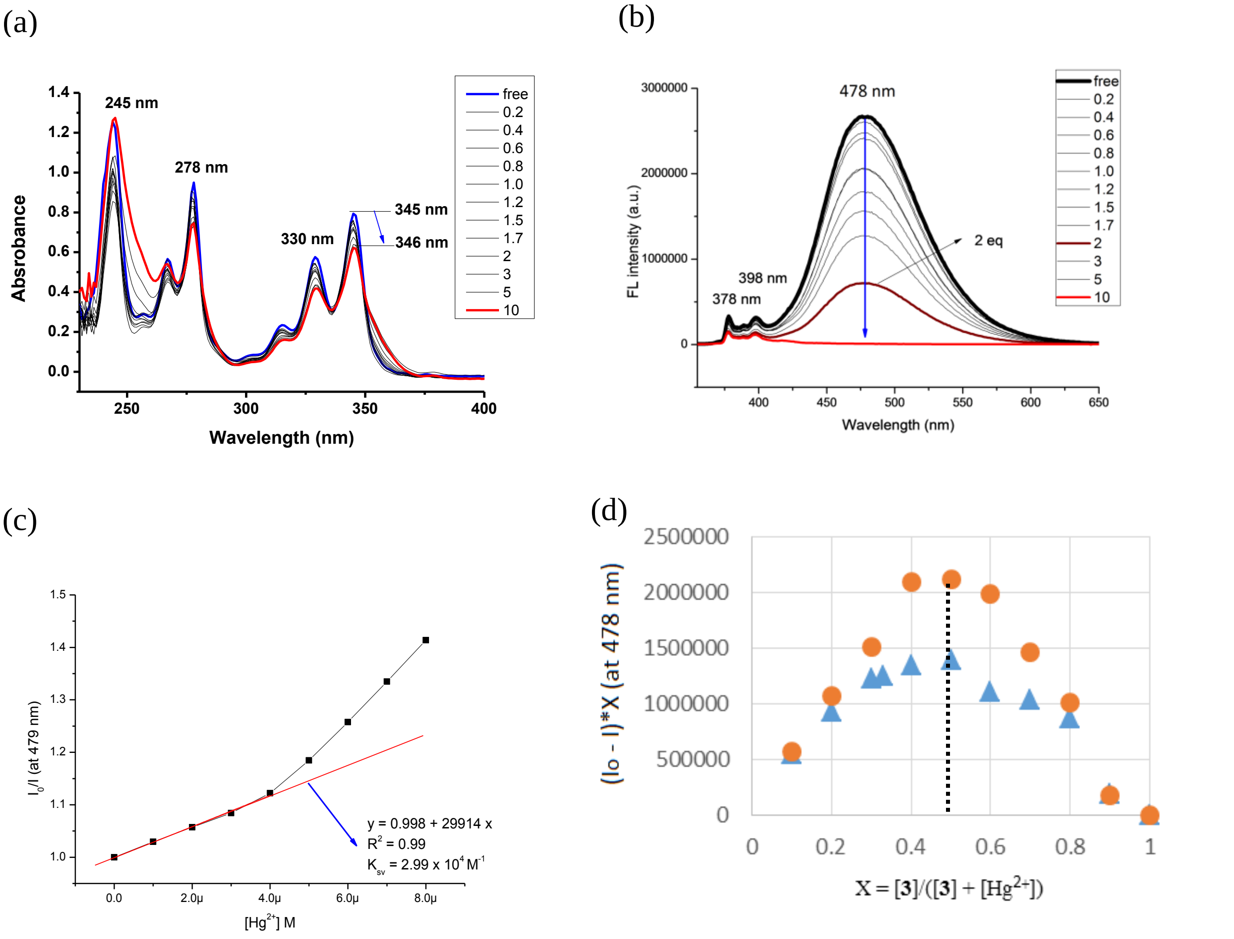

Figure S8. (a) The UV-Vis spectra, (b) the fluorescence emission spectra, (c) the Stern-Volmer plot fitting, and (d) the Job plot of ligand **3** (10 μM) with various equiv. of $Hg(ClO_4)_2$ in co-solvent $CHCl_3$/MeOH (v/v, 3:1), two sets of experimental data are overlaid.

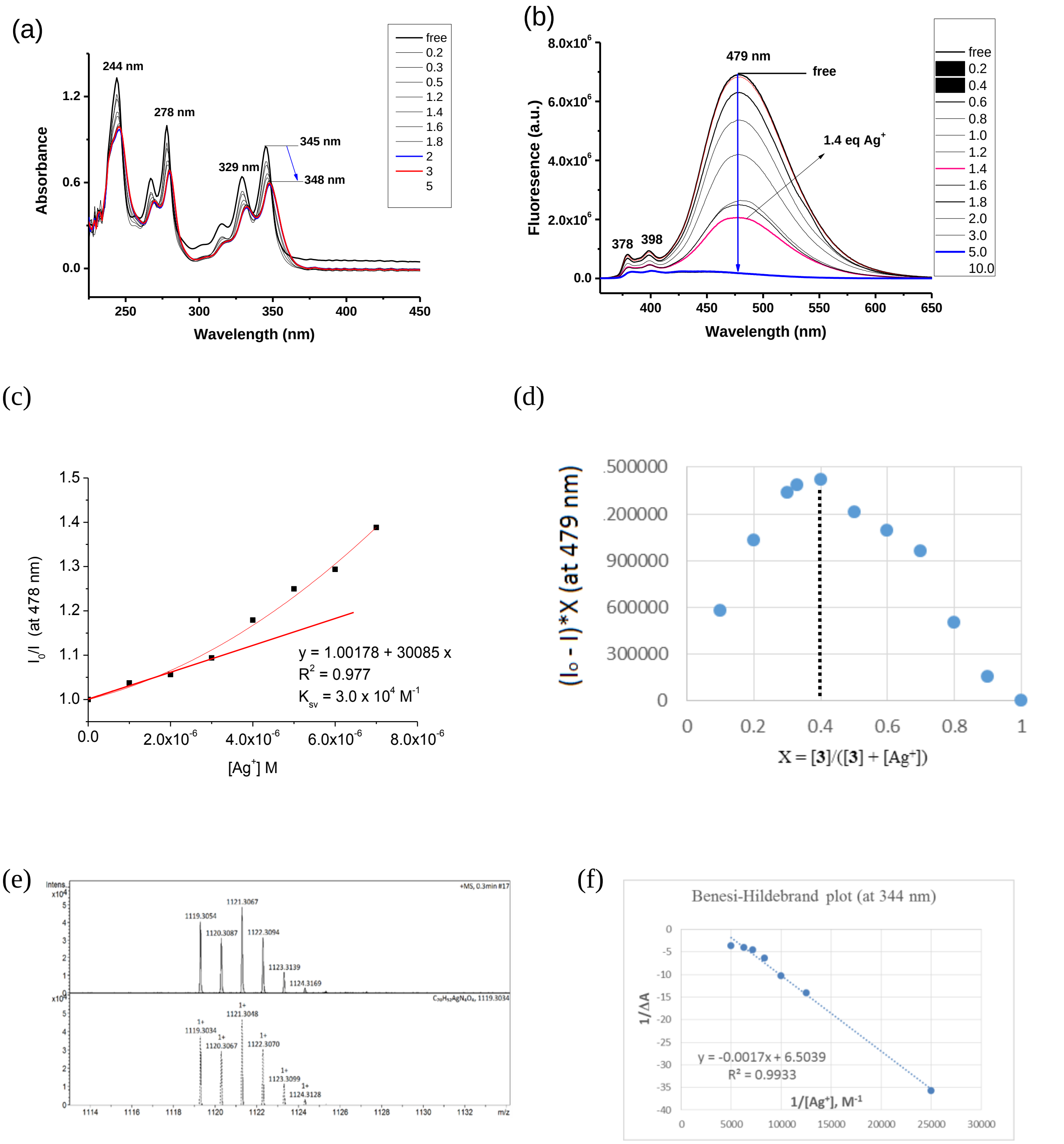

Figure S9. (a) The UV-Vis spectra, (b) the fluorescence emission spectra, (c) the Stern-Volmer plot fitting, (d) the Job plot of ligand **3** (10 μM) with various equiv. of $AgClO_4$ in co-solvent $CHCl_3$/MeOH (v/v, 3:1), (e) HRMS of a 1:1 complex of **3**•Ag^+ and (f) Benesi-Hildebrand plot of the UV-vis absorption of ligand **3** (10 μM) at 344 nm with various equiv. of $AgClO_4$ in co-solvent $CHCl_3$/MeOH (v/v, 3:1) at 298K.

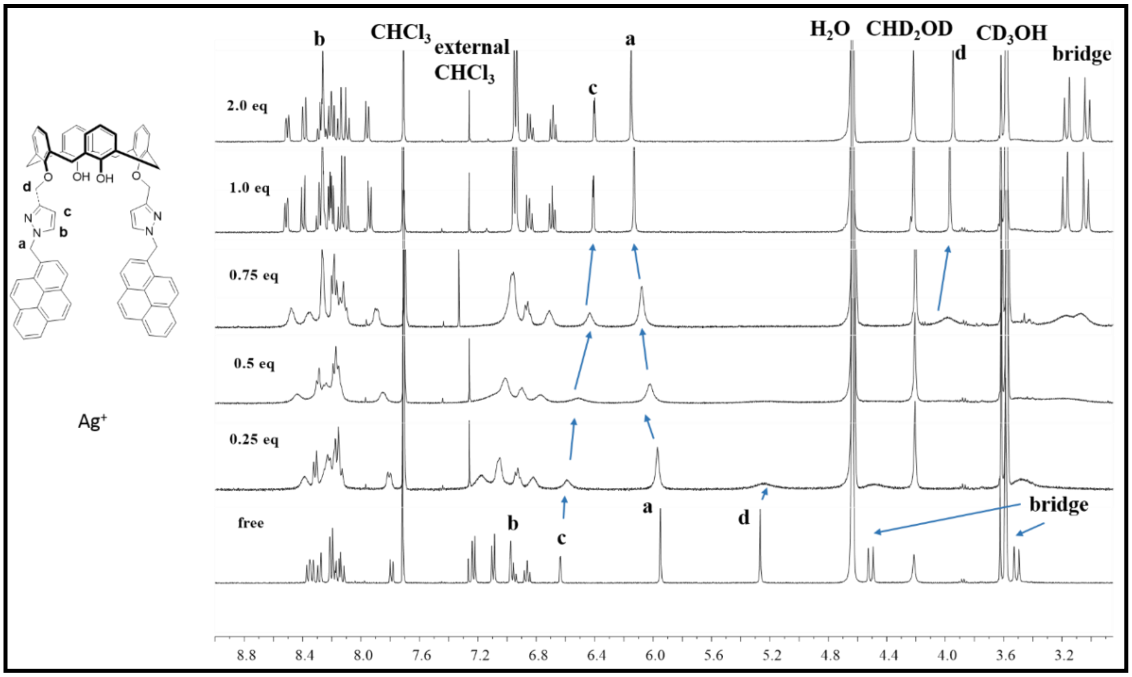

Figure S10. The ^{1}H NMR titration of ligand **3** (1.33 mM) in $CDCl_3/CD_3OH$ (v/v, 3:1) in the presence of different equiv of $AgClO_4$: (a) 0, (b) 0.25, (c) 0.5, (d) 0.75, (e) 1.0, and (f) 2.0.

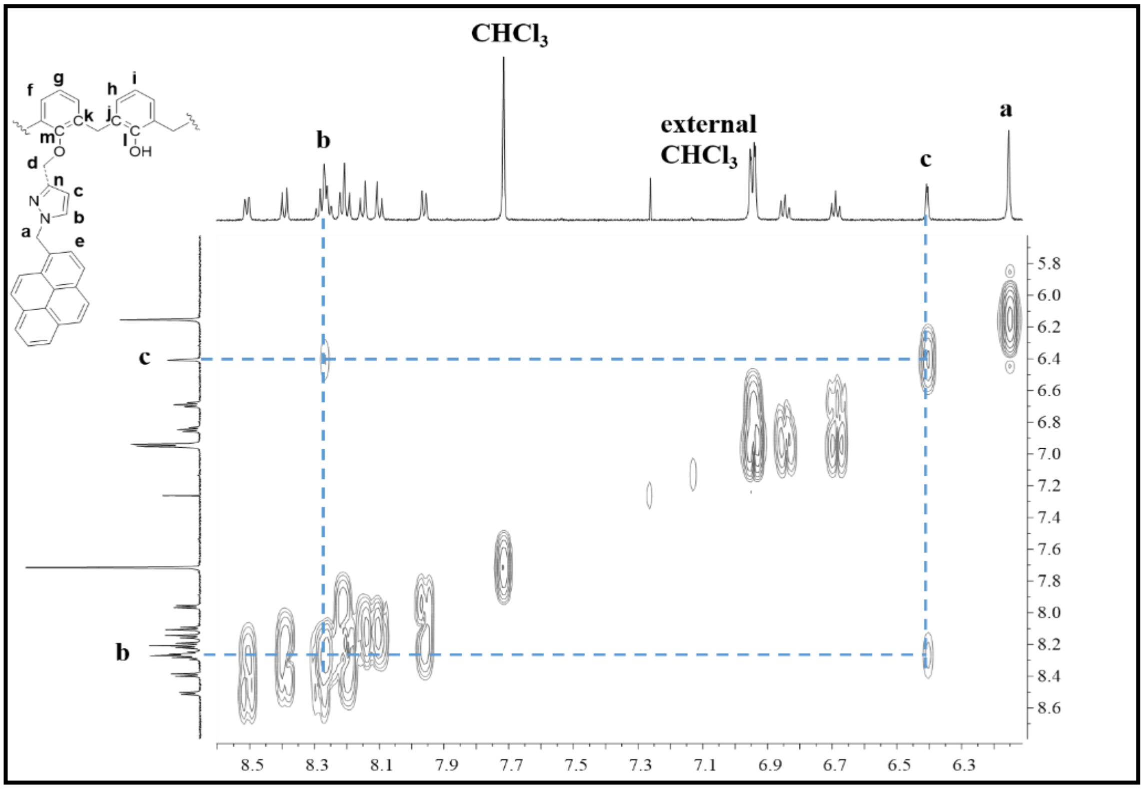

Figure S11. The H,H-COSY of ligand **3** in the presence of 2 equiv of Ag^+ in $CDCl_3/CD_3OH$ (v/v, 3:1).

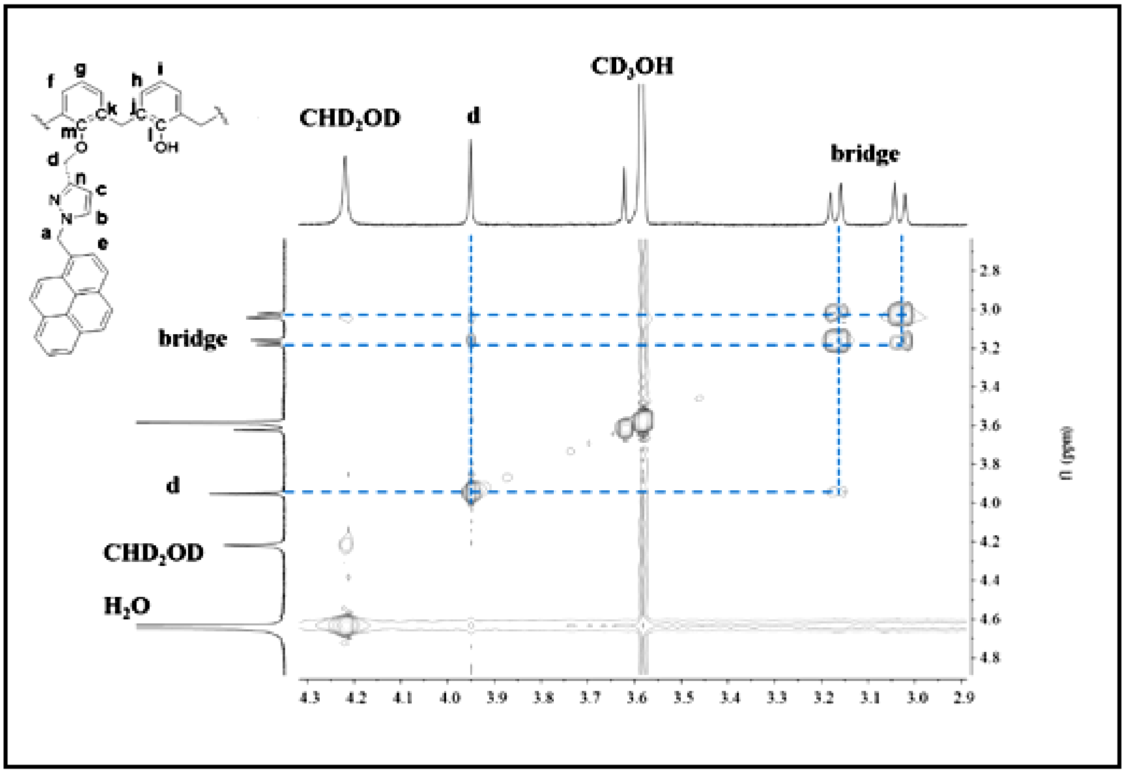

Figure S12. The 2D-NOESY spectrum of ligand **3** in the presence of 2 equiv of Ag^+ in $CDCl_3/CD_3OH$ (v/v, 3:1).

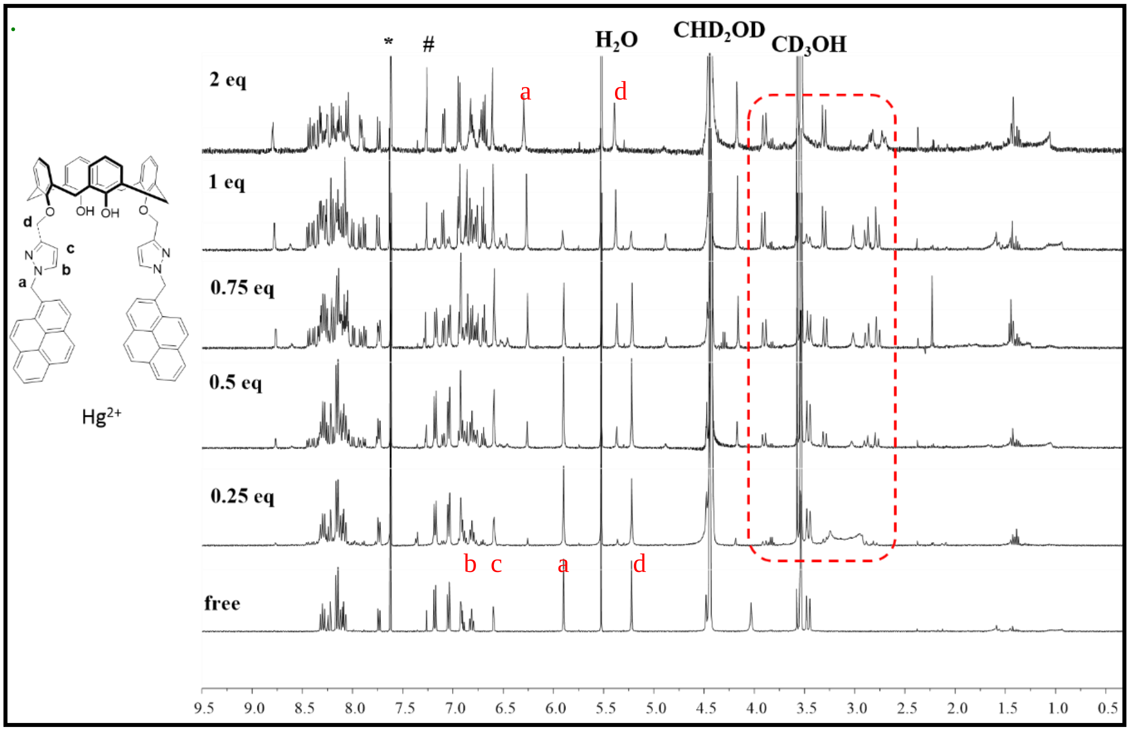

Figure S13. The ^1H NMR titration of ligand **3** (1.33 mM) in $CDCl_3/CD_3OH$ (v/v, 3:1) in the presence of different equiv of $Hg(ClO_4)_2$: (a) 0, (b) 0.25, (c) 0.5, (d) 0.75, (e) 1.0, and (f) 2.0.

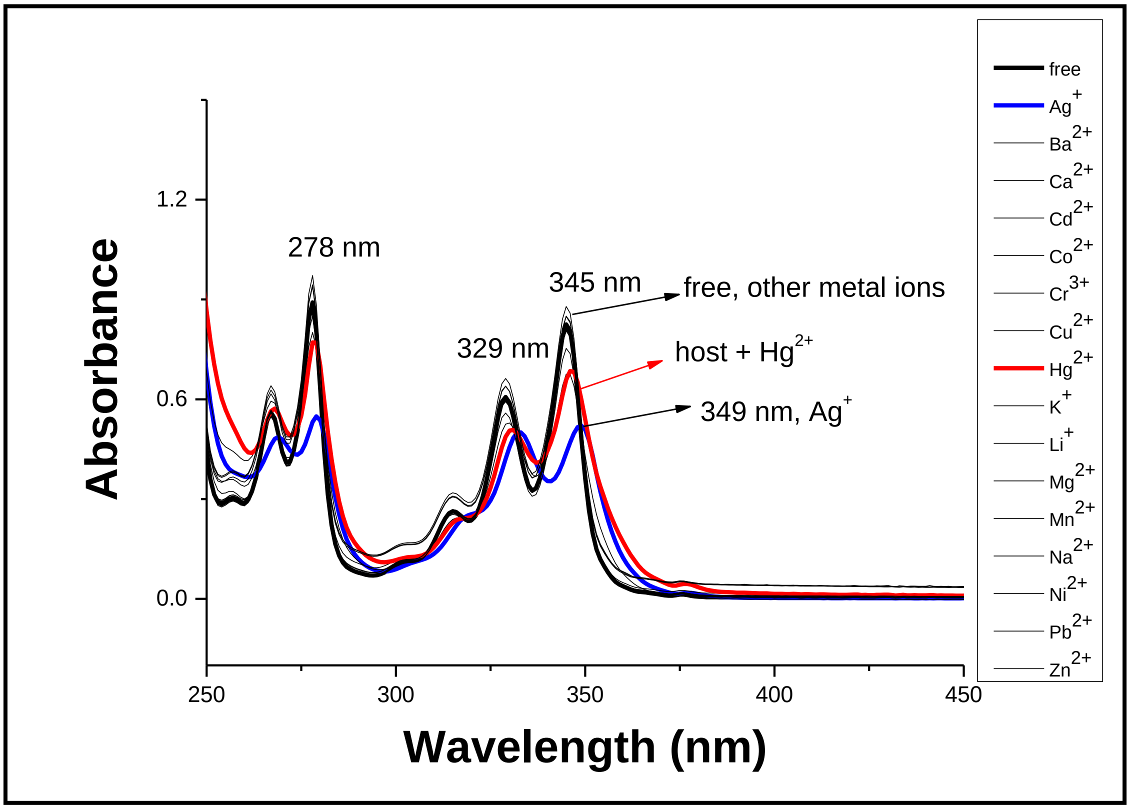

Figure S14. UV-Vis spectrum of ligand **8** (10 μM) by the addition of 10 equiv of various metal perchlorates (Ag^{+}, Ba^{2+}, Ca^{2+}, Cd^{2+}, Co^{2+}, Cr^{3+}, Cu^{2+}, Hg^{2+}, Li^{+}, Na^{+}, K^{+}, Mg^{2+}, Ni^{2+}, Pb^{2+}, and Zn^{2+}) in co-solvent $CHCl_3$/MeOH (v/v, 3:1).

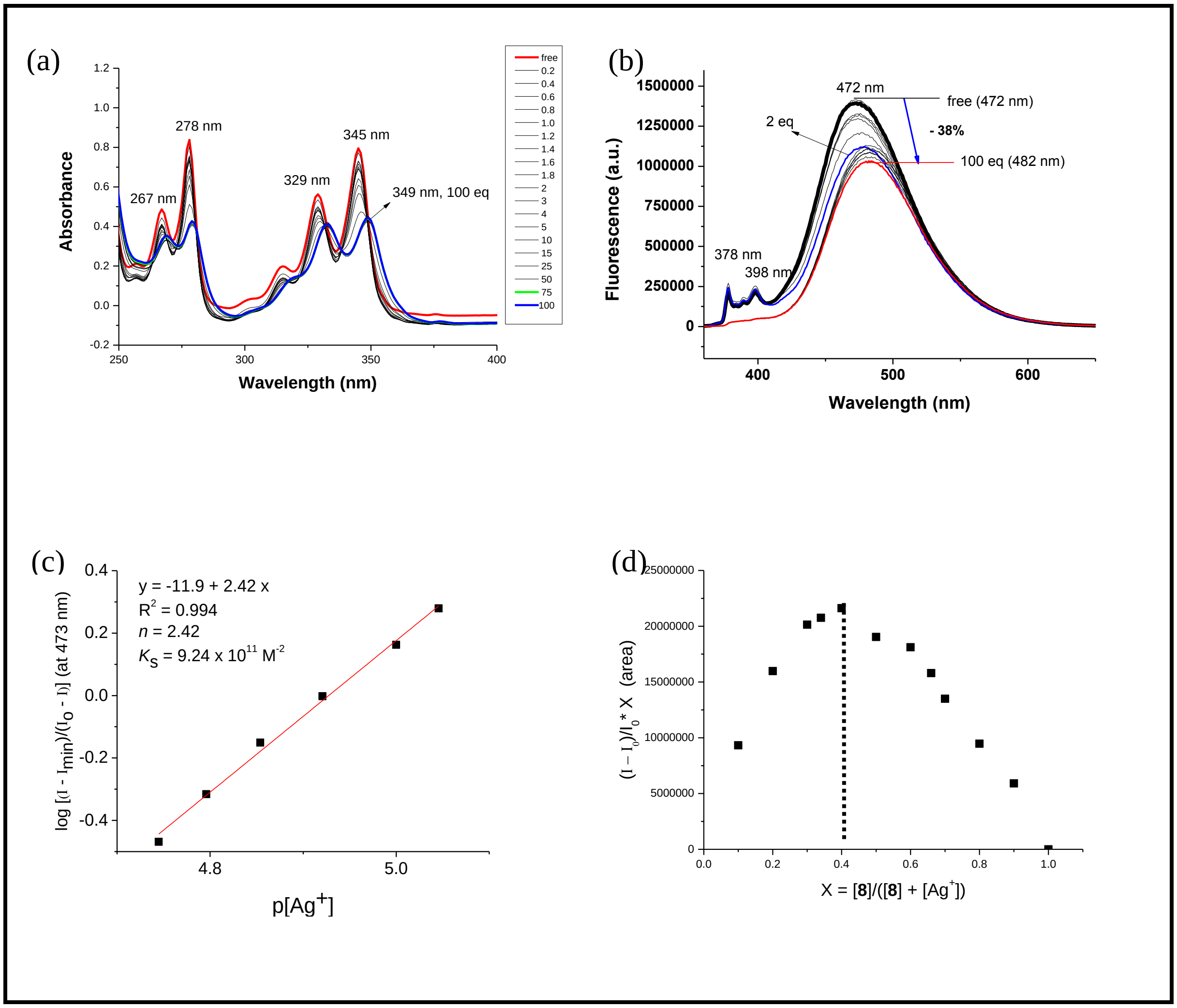

Figure S15. (a) The UV-Vis spectra, (b) the fluorescence emission spectra, (c) the Hill plot fitting, and (d) the Job plot of ligand **8** (10 μM) with various equiv of $AgClO_4$ in co-solvent $CHCl_3$/MeOH (v/v, 3:1). The excitation wavelength was 339 nm.

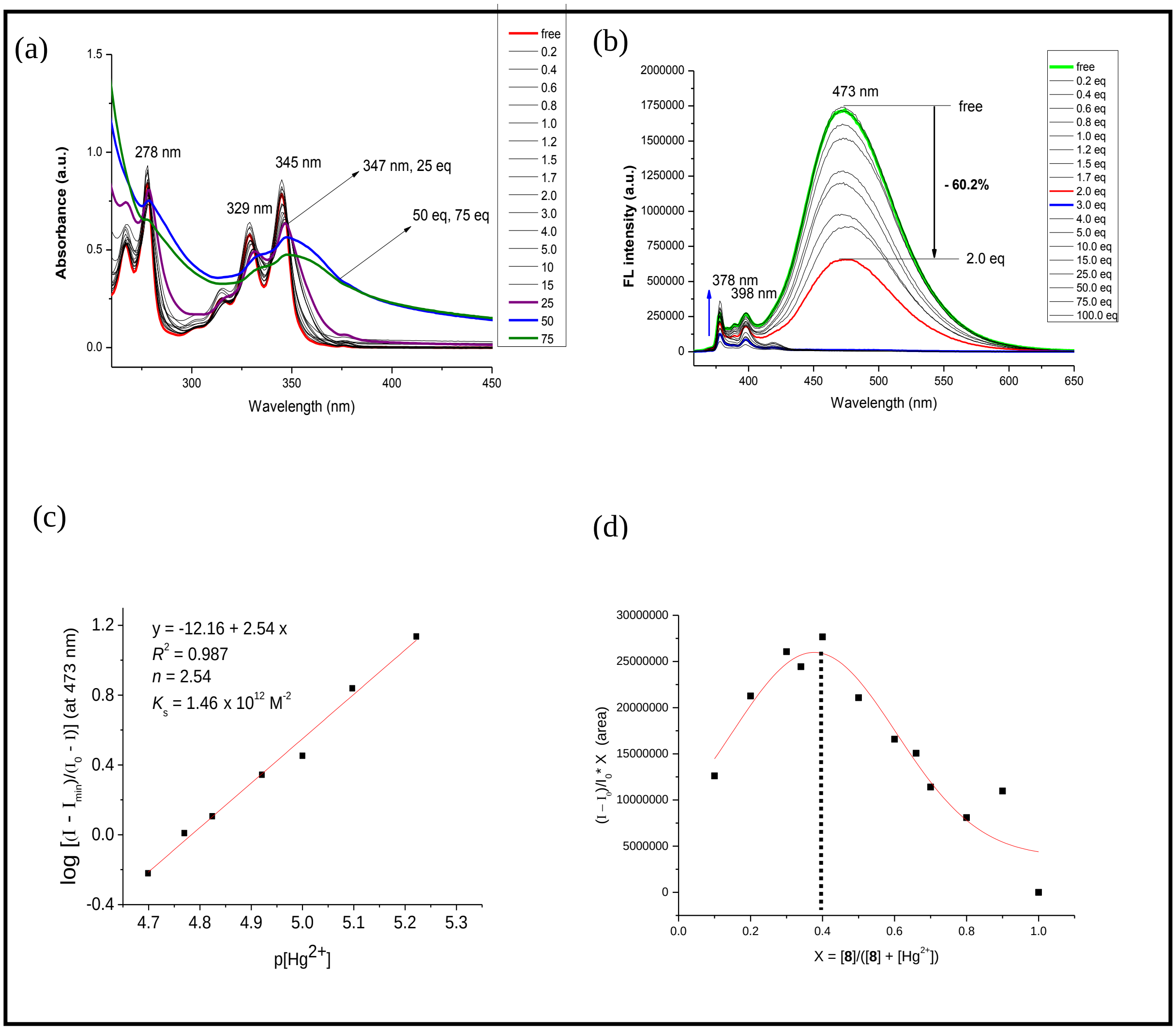

Figure S16. (a) The UV-Vis spectra, (b) the fluorescence emission spectra, (c) the Hill plot fitting, and (d) the Job plot of ligand **8** (10 μM) with various equiv of $Hg(ClO_4)_2$ in co-solvent $CHCl_3$/MeOH (v/v, 3:1).

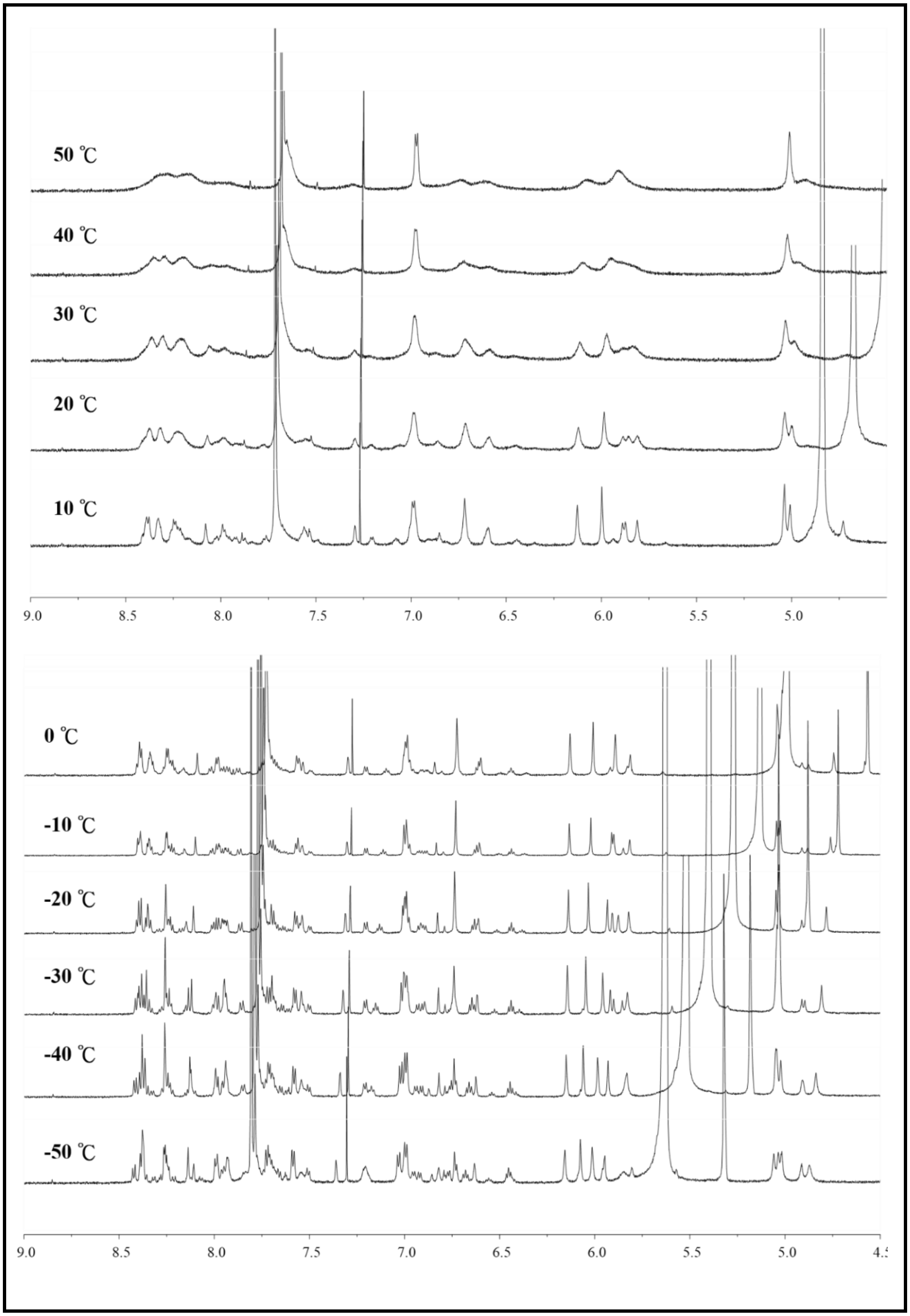

Figure S17. Variable temperature ^{1}H NMR spectra (-50 to 50 ^{o}C, 600 MHz) of ligand **8** (1.33 mM) in $CDCl_3/CD_3OH$ (v/v, 3:1) with 1 equiv of $AgClO_4$.

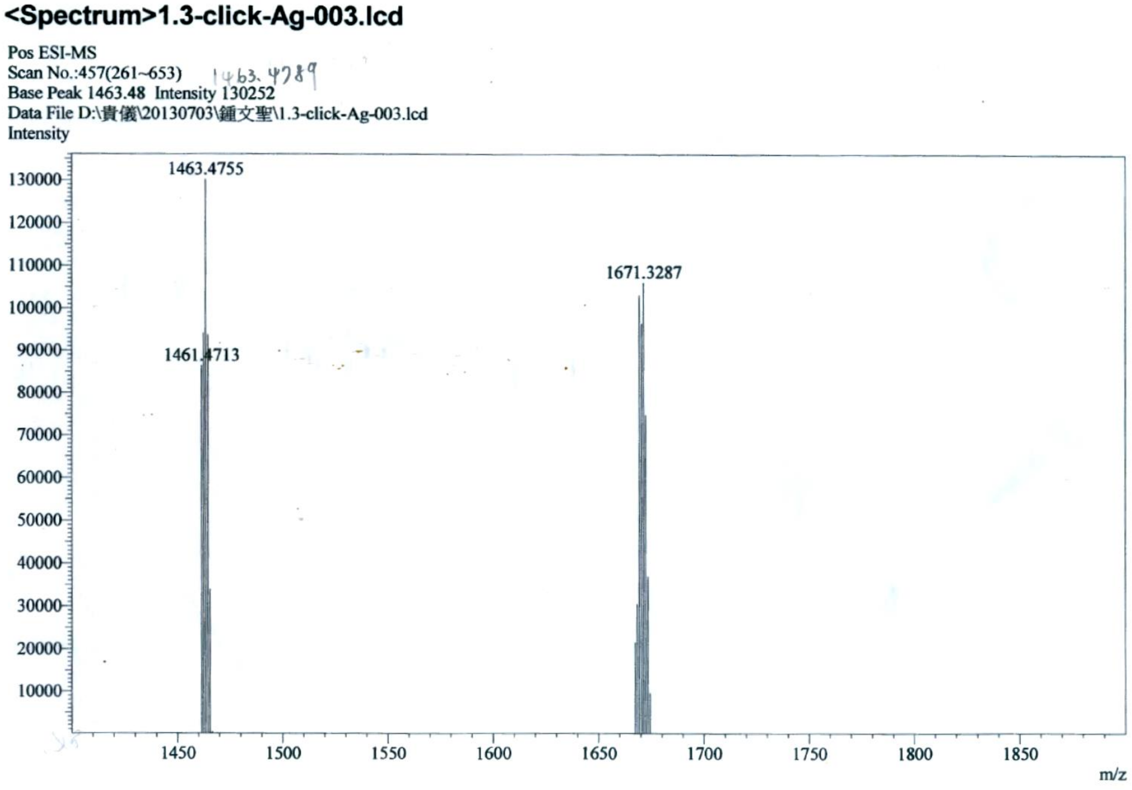

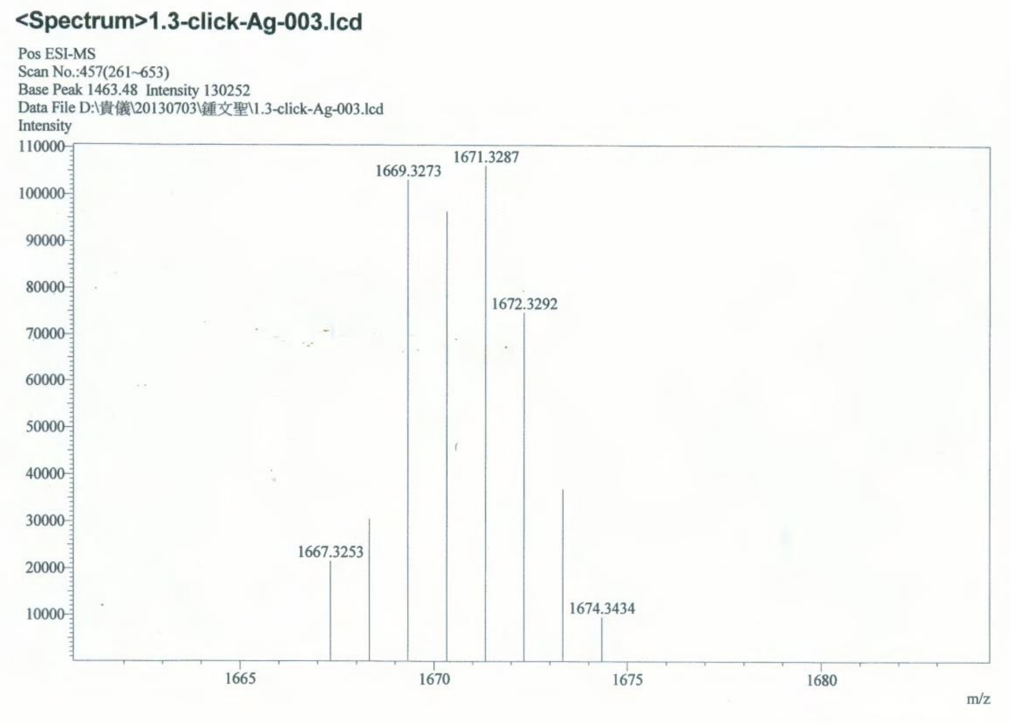

Figure S18. The ESI-MS data of the complex **8**•$(Ag^{+})_2$•ClO_4^{-}.

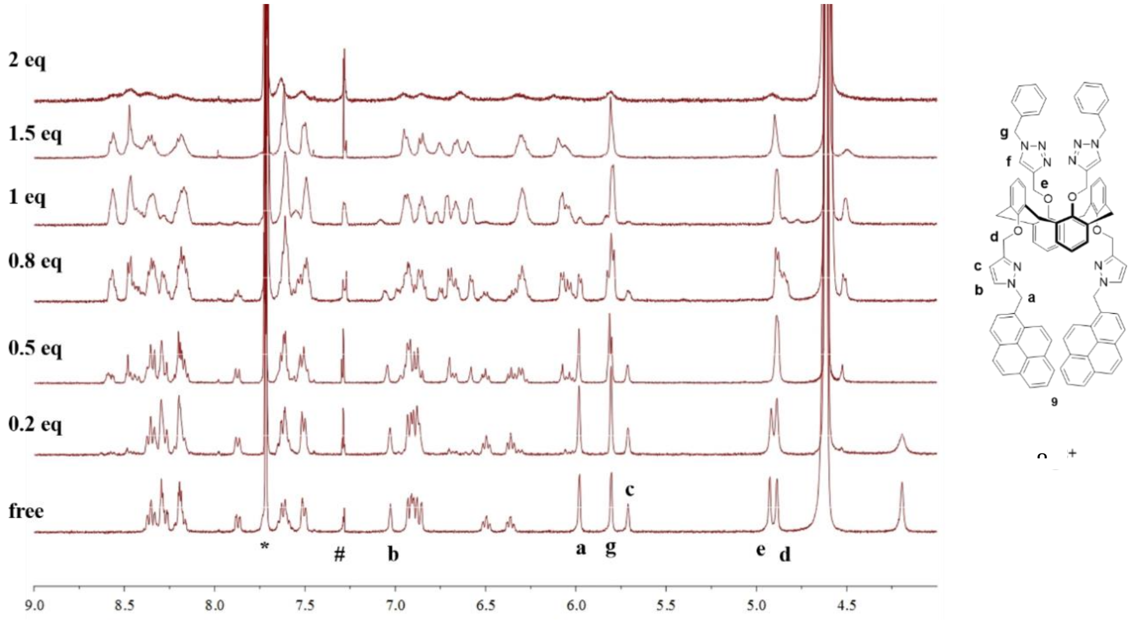

Figure S19. The ^{1}H NMR titration spectra of ligand **8** (1.33 mM) in the presence of different equiv of $Hg(ClO_4)_2$ in $CDCl_3/CD_3OH$ (v/v, 3:1); where * denotes residual $CHCl_3$ and # denotes external $CHCl_3$.
